# Transcription and translation of the *sigG* gene is tuned for proper execution of the switch from early to late gene expression in the developing *Bacillus subtilis* spore

**DOI:** 10.1371/journal.pgen.1007350

**Published:** 2018-04-27

**Authors:** Elizabeth B. Mearls, Jacquelin Jackter, Jennifer M. Colquhoun, Veronica Farmer, Allison J. Matthews, Laura S. Murphy, Colleen Fenton, Amy H. Camp

**Affiliations:** 1 Department of Biological Sciences, Mount Holyoke College, South Hadley, MA, USA; 2 Department of Biological Sciences, Lehigh University, Bethlehem, PA, USA; Max Planck Institute for Terrestrial Microbiology, GERMANY

## Abstract

A cascade of alternative sigma factors directs developmental gene expression during spore formation by the bacterium *Bacillus subtilis*. As the spore develops, a tightly regulated switch occurs in which the early-acting sigma factor σ^F^ is replaced by the late-acting sigma factor σ^G^. The gene encoding σ^G^ (*sigG*) is transcribed by σ^F^ and by σ^G^ itself in an autoregulatory loop; yet σ^G^ activity is not detected until σ^F^-dependent gene expression is complete. This separation in σ^F^ and σ^G^ activities has been suggested to be due at least in part to a poorly understood intercellular checkpoint pathway that delays *sigG* expression by σ^F^. Here we report the results of a careful examination of *sigG* expression during sporulation. Unexpectedly, our findings argue against the existence of a regulatory mechanism to delay *sigG* transcription by σ^F^ and instead support a model in which *sigG* is transcribed by σ^F^ with normal timing, but at levels that are very low. This low-level expression of *sigG* is the consequence of several intrinsic features of the *sigG* regulatory and coding sequence—promoter spacing, secondary structure potential of the mRNA, and start codon identity—that dampen its transcription and translation. Especially notable is the presence of a conserved hairpin in the 5’ leader sequence of the *sigG* mRNA that occludes the ribosome-binding site, reducing translation by up to 4-fold. Finally, we demonstrate that misexpression of *sigG* from regulatory and coding sequences lacking these features triggers premature σ^G^ activity in the forespore during sporulation, as well as inappropriate σ^G^ activity during vegetative growth. Altogether, these data indicate that transcription and translation of the *sigG* gene is tuned to prevent vegetative expression of σ^G^ and to ensure the precise timing of the switch from σ^F^ to σ^G^ in the developing spore.

## Author summary

Global changes in gene expression occur during normal cellular growth and development, as well as during cancer cell transformation and bacterial pathogenesis. In this study we have investigated the molecular mechanisms that drive the switch from early to late developmental gene expression during spore formation by the model bacterium *Bacillus subtilis*. At early times, gene expression in the developing spore is directed by the transcription factor σ^F^; at later times σ^F^ is replaced by σ^G^. An important, yet poorly understood aspect of this σ^F^-to-σ^G^ transition is how σ^G^ activation is delayed until the early, σ^F^-directed phase of gene expression is complete. Here we have carefully examined expression of the gene encoding σ^G^, *sigG*, and found that its transcription and translation are ordinarily dampened by several features of its regulatory and coding sequences. Moreover, we have found that this “tuning” of *sigG* expression is required for proper timing of the switch to σ^G^. These results reframe our understanding of how *sigG* is regulated during *B*. *subtilis* sporulation and, more broadly, advance our understanding of how global changes in gene expression can be precisely executed at the molecular/genetic level.

## Introduction

**Fig 1:**
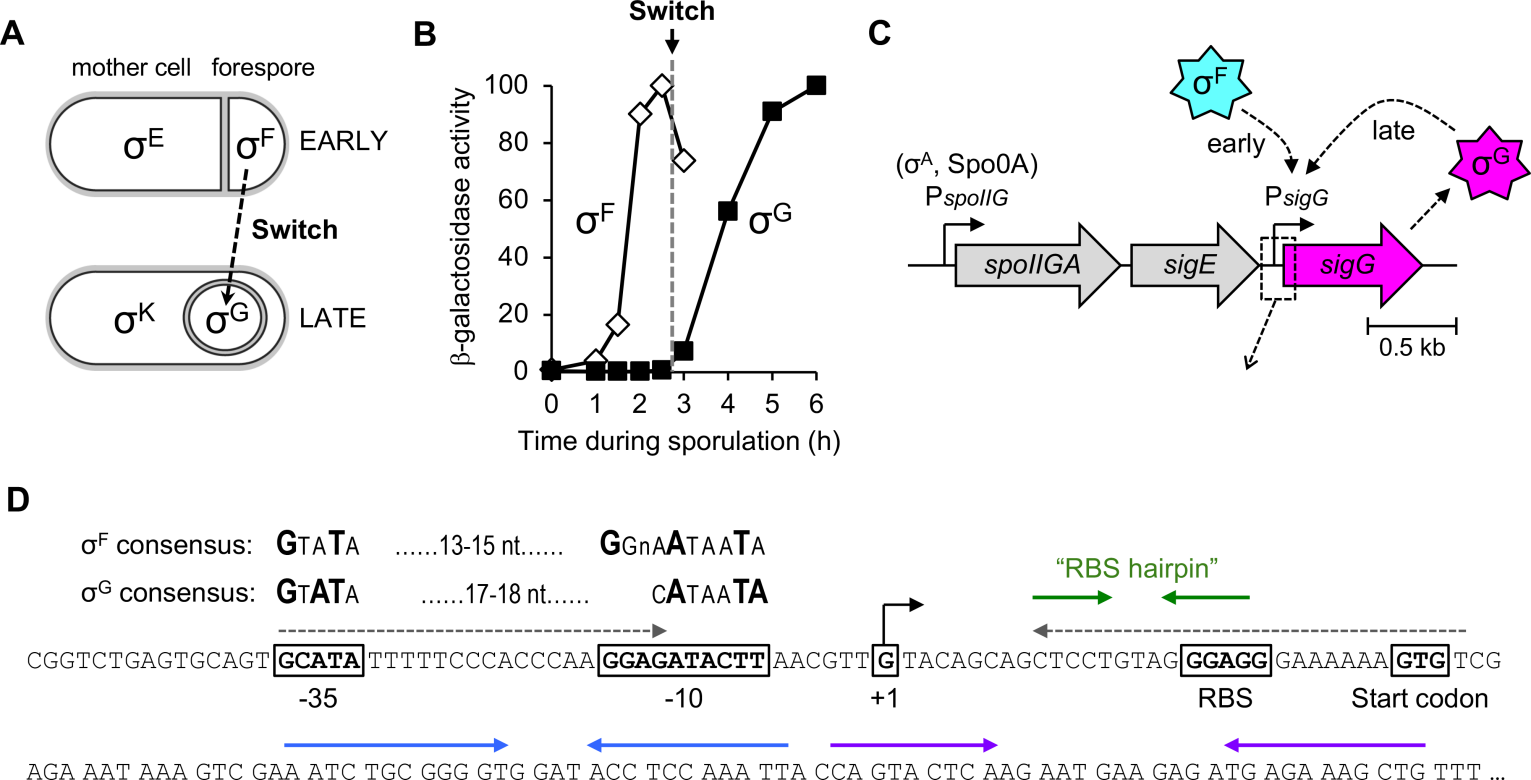
Expression of the *sigG* gene and the switch from σ^F^ to σ^G^ during *B*. *subtilis* sporulation. **(A)** Cartoon depicting the sigma factors that govern gene expression during early (top) and late (bottom) stages of *B*. *subtilis* sporulation. At early times, the sigma factors σ^F^ and σ^E^ control gene expression in the forespore and mother cell, respectively. At later times, after forespore engulfment by the mother cell, σ^F^ is replaced by σ^G^ and σ^E^ is replaced by σ^K^. The switch from σ^F^ to σ^G^ is indicated by a dashed arrow. **(B)** The switch from σ^F^ to σ^G^. The σ^F^-dependent expression of a P_*spoIIQ*_*-lacZ* reporter (open diamonds) and σ^G^-dependent expression of a P_*sspB*_*-lacZ* reporter (closed squares) were monitored during sporulation of wild type cells. (Strains AHB881 and AHB317, respectively.) All *lacZ* reporters used in this study, unless otherwise noted, were at the non-essential *amyE* locus. β-Galactosidase activity is reported as the percent maximum for each. The timing of the σ^F^-to-σ^G^ switch, between sporulation hours 2.5 and 3, is indicated by a dashed gray line. **(C)** Cartoon depicting the *sigG* gene in its native chromosomal location downstream of the *spoIIGA-sigE* operon, and our new, simplified model for its transcriptional regulation by σ^F^ at early times and σ^G^ itself at late times. Dashed box/arrow indicates region depicted in (D). **(D)** Sequence of the *sigG* upstream regulatory region and 5’ coding region (codons 1–28). The -35 and -10 *sigG* promoter elements [9] are boxed, with the consensus σ^F^ and σ^G^-recognized sequences shown above [38]. Also boxed are the *sigG* +1 transcription start site, RBS, and GTG start codon. Gray dashed arrows indicate complementary sequences predicted to form a hairpin structure that blocks translation of read-through transcripts originating from P_*spoIIG*_ [52]; note that this hairpin cannot form in transcripts originating from P_*sigG*_. Green, blue, and purple solid arrows indicate complementary sequences predicted to form hairpin secondary structures in mRNA transcripts originating from P_*sigG*_.

Cells across all domains of life alter their phenotypes through global changes in gene expression. In bacteria, global changes in gene expression drive phenotypic changes critical for growth, development, and pathogenesis. For example, the ability of the human pathogen *Chlamydia trachomatis* to progress through its infectious cycle requires sequential transitions between three stage-specific networks of gene regulation [1]. Here, we study a switch in gene expression that occurs during the developmental process of spore formation by the soil bacterium *Bacillus subtilis*, a premier model system for studies of regulation [2,3]. At the onset of *B*. *subtilis* sporulation, which is triggered by nutrient depletion, the rod shaped bacterial cell divides asymmetrically, resulting in two daughter cells of unequal size and fate. The smaller “forespore” becomes the mature, dormant spore, while the larger “mother cell” aids the development of the forespore but ultimately dies. Initially these two cells lie side-by-side; subsequently, the mother cell membranes migrate around the forespore in a process called engulfment, resulting in the forespore being pinched off as a free protoplast within the mother cell cytoplasm (Fig 1A). After engulfment, the forespore is encased in a protective peptidoglycan cortex and protein coat, and is then released into the environment as a mature spore upon lysis of the mother cell.

The morphological events of sporulation are orchestrated by a complex gene regulatory network that coordinates the expression of hundreds of sporulation genes at the right time and in the right cell [3,4]. This gene regulatory network operates primarily to ensure the sequential and compartment-specific appearance of four sporulation sigma (σ) factors—σ^F^, σ^E^, σ^G^, and σ^K^—that bind and impart promoter specificity upon core RNA polymerase (RNAP). Early in sporulation, following asymmetric division, σ^F^ and σ^E^ direct gene expression in the forespore and mother cell, respectively. Later, after the completion of engulfment, σ^G^ takes over for σ^F^ in the forespore and σ^K^ replaces σ^E^ in the mother cell (Fig 1A). These two transitions, from σ^F^- to σ^G^-directed gene expression in the forespore and from σ^E^- to σ^K^-directed gene expression in the mother cell, are tightly regulated such that no temporal overlap between the activities of the early and late sigma factors can be detected [5]. However, the molecular mechanisms that control these global transitions in gene expression with such precision are not well understood.

In this study, we sought to identify molecular mechanisms that help to orchestrate the switch from σ^F^ to σ^G^ in the *B*. *subtilis* forespore (Fig 1A and 1B). The current model for the σ^F^-to-σ^G^ switch, summarized here, is based on literature spanning several decades. To begin, σ^F^ is activated in the forespore soon after asymmetric cell division via a complex, but relatively well-characterized regulatory circuit [6]. In turn, σ^F^ directs the transcription of genes required for early forespore development as well as the gene *sigG* (previously *spoIIIG*) that encodes σ^G^ [7–9]. Any σ^G^ produced at these early times remains inactive; only after forespore engulfment is complete does σ^G^ become active and replace σ^F^. The deactivation of σ^F^ is poorly understood, but involves the small protein Fin (previously YabK) as well as other unidentified mechanisms [10,11]. The subsequent activity of σ^G^ requires an intercellular channel apparatus comprised of the mother cell proteins SpoIIIAA-AH and forespore protein SpoIIQ (reviewed in [12]). Interestingly, this SpoIIIAA-AH•SpoIIQ channel does not specifically regulate σ^G^, but instead is required more generally to maintain forespore physiology and to support any forespore gene expression at late times, even that engineered to be directed by a heterologous phage RNAP [13,14]. Finally, once active, σ^G^ directs the transcription of genes required for late forespore development, as well as its own gene in an autoregulatory loop [9], thus locking in the transition to the late program of developmental gene expression in the forespore.

A major unanswered question regarding the switch from early to late gene expression in the forespore is how σ^G^ activity is delayed until the early, σ^F^-directed phase of gene expression is complete. One protein that has been implicated in controlling early σ^G^ activity is the anti-sigma factor CsfB (also called Gin), which is expressed under the control of σ^F^ at early times and is a potent antagonist of σ^G^ [15–18]. Deletion of *csfB* has been reported to cause premature activation of σ^G^ in a subset of sporulating cells [16]. But other studies have concluded that *ΔcsfB* does not alter the level nor timing of peak σ^G^ activity [19], and *ΔcsfB* cells display no defect in spore formation [15]. These results suggest that CsfB-independent mechanisms must also be in place to restrict σ^G^ activity at early times.

A second possible explanation for the delay in σ^G^ activation comes from reports that the transcription of *sigG* by σ^F^ is delayed by up to an hour relative to other σ^F^ target genes [20,21]. In addition, σ^F^-dependent transcription of *sigG*, unlike that of other σ^F^ target genes, has been reported to require the forespore membrane protein SpoIIQ and the early-acting mother cell sigma factor σ^E^ [20,22,23]. These findings have led to speculation that a signaling pathway, perhaps involving SpoIIQ, specifically couples σ^F^-dependent *sigG* transcription to the activation of σ^E^ in the mother cell [2,24]. Satisfyingly, a checkpoint mechanism such as this would account for the observed delay in *sigG* expression, given that both *spoIIQ* expression and σ^E^ activation require the earlier activity of σ^F^ [25–27]. To test this idea, several groups have monitored the timing of σ^G^ activation during sporulation of strains engineered to express *sigG* under the control of a more typical “early” σ^F^-target promoter. The majority found no evidence of premature σ^G^ activity in such engineered strains [17,19,28], although one study reported that if *sigG* expression was boosted (by inserting three copies of the engineered *sigG* construct), inappropriate σ^G^ activity could be detected at early times [14]. This may hint at the importance of *sigG* expression levels, in addition to timing, in dictating the onset of σ^G^ activity. Overall, however, the regulation of *sigG* expression and its impact upon the timing of σ^G^ activity remains an open question.

Here we report the results of a careful examination of *sigG* expression that calls into question long-standing assumptions about its regulation. We argue here that *sigG* transcription by σ^F^ is not delayed, nor does it require a specific intercellular signaling pathway originating in the mother cell. Instead, we propose a simpler model (Fig 1C) in which *sigG* is first transcribed by σ^F^ with normal timing, but at levels that are very low and, as such, difficult to detect. Subsequently, the majority of *sigG* transcription occurs later under the control of σ^G^ itself. We present evidence that the low-level expression of *sigG* at early times is a consequence of four intrinsic features of the *sigG* regulatory and coding sequences that dampen its transcription and translation: suboptimal spacing between the -10 and -35 *sigG* promoter elements, a suboptimal translational start codon, a hairpin in the 5’ leader sequence of the *sigG* mRNA that occludes the ribosome-binding site (RBS), and a suboptimal 5’ coding sequence. Finally, we demonstrate that misexpression of *sigG* from regulatory sequences lacking these features results in inappropriate σ^G^ activity during vegetative growth and premature σ^G^ activity in the forespore during sporulation.

## Results

### *sigG* expression can be detected before and after the switch from σ^F^ to σ^G^

Before examining *sigG* expression during sporulation, we first determined the timing of the switch from σ^F^ to σ^G^ under our sporulation conditions using *lacZ* reporters fused to promoters under the exclusive control of each sigma factor. (All *lacZ* reporter genes in this study were integrated at the non-essential *amyE* locus.) As shown in Fig 1B, β-galactosidase production from a *lacZ* reporter fused to the σ^F^-dependent *spoIIQ* promoter (P_*spoIIQ*_) [27] was first detected above background at approximately hour 1.5 of sporulation, peaked at hour 2.5, and declined thereafter. In contrast, a *lacZ* reporter fused to the σ^G^-dependent *sspB* promoter (P_*sspB*_) [29] turned on at hour 3 of sporulation, after which time β-galactosidase production continued to rise through hour 5 (Fig 1B). Together, these findings indicate that under our sporulation conditions, σ^F^ is active from hours 1.5–2.5, σ^G^ is active from hours 3–6, and the switch from σ^F^ to σ^G^ occurs between hours 2.5–3.

**Fig 2:**
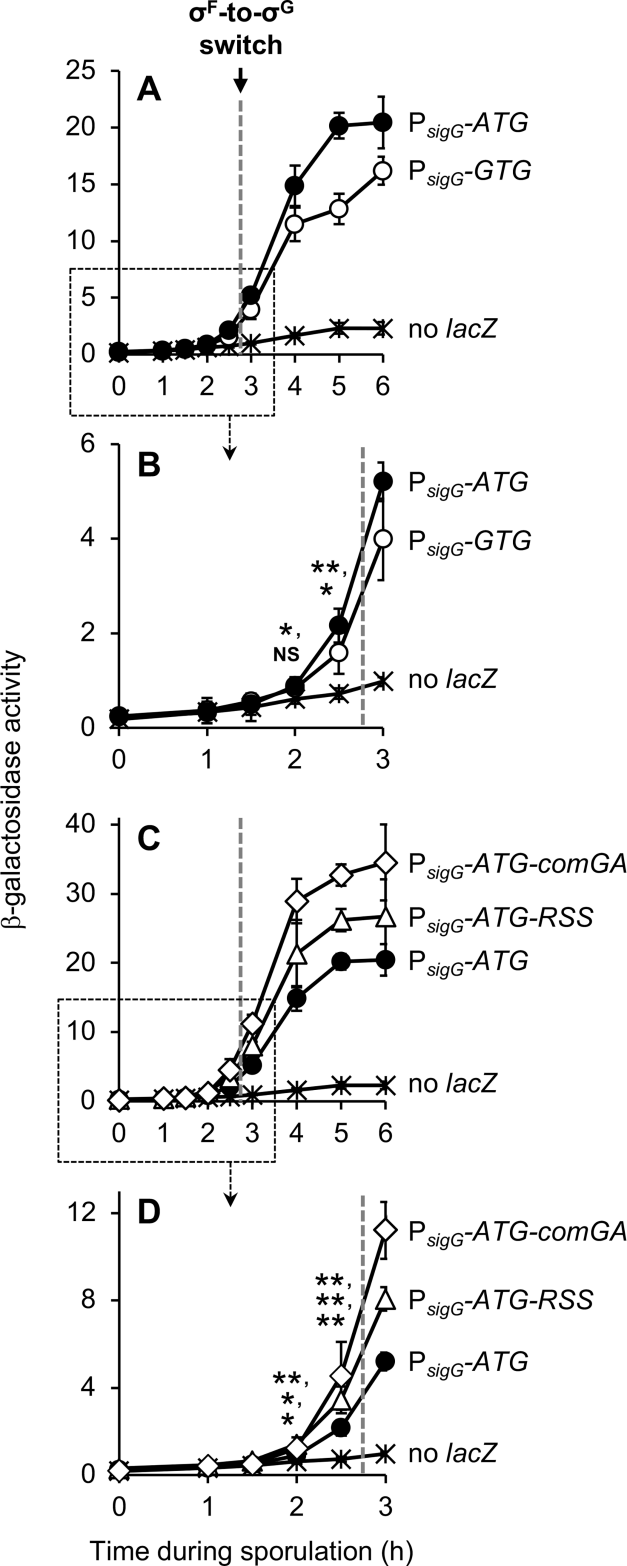
P_*sigG*_ activity is detected both before and after the switch from σ^F^ to σ^G^. **(A, B)** The activities of P_*sigG*_ reporters harboring the native GTG start codon, P_*sigG*_*-sigG*^*1-28*^*-lacZ* (P_*sigG*_*-GTG*; open circles) or engineered to harbor an ATG start codon, P_*sigG*_*-ATG-sigG*^*2-28*^*-lacZ* (P_*sigG*_*-ATG*; closed circles), were measured during a time course of sporulation. (Strains EBM177 and EBM175, respectively.) Background β-galactosidase activity was measured in a strain without a *lacZ* reporter, PY79 (no *lacZ*; asterisks). Note that (B) provides a zoomed view and statistical significance of early sporulation data points from the boxed area of (A). **(C, D)** Strains harboring P_*sigG*_ reporters with altered *sigG* 5’ coding sequence, one engineered to reduce secondary structure (RSS) in *sigG* codons 2–28, P_*sigG*_*-ATG-*^*RSS*^*sigG*^*2-28*^*-lacZ* (P_*sigG*_*-ATG-RSS*; open triangles), and another harboring *comGA* codons 2–8 in place of *sigG* codons 2–28, P_*sigG*_*-ATG-comGA*^*2-8*^*-lacZ* (P_*sigG*_*-ATG-comGA*; open diamonds), were monitored for β-galactosidase production during sporulation. (Strains EBM237 and JJB31, respectively.) Data for the P_*sigG*_*-ATG-sigG*^*2-28*^*-lacZ* (P_*sigG*_*-ATG*; closed circles) strain (EBM175) and PY79 (no *lacZ*, asterisks), the same as shown in (A) and (B), are also included for reference. Note that (D) provides a zoomed view and statistical significance of early sporulation data points from the boxed area of (C). The timing of the σ^F^-to-σ^G^ switch, between sporulation hours 2.5 and 3, is indicated in each panel by a dashed gray line. For all panels, error bars indicate ± standard deviations based on three independent experiments. *p < 0.05, **p < 0.01, NS not significant, Student’s *t*-test.

To monitor *sigG* expression, we fused the entire *sigG* regulatory region (-203 to +114, in reference to a +1 transcription start site [9]), including the *sigG* promoter (P_*sigG*_), ribosome binding site (RBS), and first 28 codons, in-frame to *lacZ* (see Fig 1D). (In this and other experiments in this study, unless otherwise indicated, the native *sigG* gene was left intact, under the control of its wild-type regulatory sequences.) As shown in Fig 2A and 2B, expression of this P_*sigG*_*-sigG*^*1-28*^*-lacZ* reporter gene was first detectable above background at very low, but statistically significant levels at hour 2.5 of sporulation, after which time its expression increased substantially. This profile indicates that the majority of *sigG* expression occurs during times when σ^G^ is active (hours 3–6), but that very low levels of *sigG* expression can be detected at slightly earlier times (hour 2.5).

To improve our ability to detect low levels of P_*sigG*_ transcription that may be occurring at early times of sporulation, we altered our *lacZ* reporter gene to optimize translation. First, we replaced the native *sigG* translation start codon, GUG (GTG), with the more efficiently utilized start codon AUG (ATG) [30]. Consistent with modestly improved translation, the modified reporter gene displayed a 1.3-fold increase in β-galactosidase activity at sporulation hours 2.5–6 relative to the original reporter gene harboring the native GTG start codon (Fig 2A). Notably, introduction of the ATG start codon also permitted the detection of weak, but statistically significant β-galactosidase activity at hour 2 of sporulation (Fig 2B). Given that the native *sigG* gene in this experiment was expressed from its unaltered wild-type regulatory sequences (including its suboptimal GTG start codon), any potential further amplification of *sigG* expression that might have resulted from enhanced σ^G^ autoregulation (i.e. if GTG had been replaced with ATG at the native site) was not measured in this assay.

To further optimize translation, we turned our attention to the remaining 27 *sigG* codons (codons 2–28) fused in frame to *lacZ*. The 5’ coding region can be a major determinant of translation efficiency, likely due to the presence of rare codons and/or the propensity of this region to form RNA secondary structures that impede translation initiation (reviewed in [31]). Notably, the *sigG* 5’ coding sequence has the potential to form several hairpin structures (Fig 1D, indicated by blue and purple arrows). As a first strategy to optimize the 5’ coding region of our P_*sigG*_ reporter, we utilized the mRNA Optimizer tool [32] to redesign *sigG* codons 2–28 such that the potential for secondary structure was minimized without altering the encoded protein. (See S1 Fig for the optimized sequence and its secondary structure potential; also, we note that this tool does not optimize for species-specific biases in codon usage.) In a second approach, we altogether replaced *sigG* codons 2–28 with codons 2–8 of the highly expressed *B*. *subtilis* gene *comGA*, a strategy that has been demonstrated to significantly improve translation of another reporter gene [33]. As shown in Fig 2C and 2D, the resulting P_*sigG*_ reporters with ATG start codons and optimized 5’ coding sequences were expressed more robustly (~1.5-fold and ~2-fold, respectively) at sporulation hours 2–6 relative to the reporter harboring only the ATG alteration. Together, this data indicates that *sigG* translation is ordinarily dampened by a suboptimal start codon and suboptimal 5’ coding sequence. Moreover, the improved reporter gene expression supports the conclusion that P_*sigG*_ is transcriptionally active both before and after the switch from σ^F^ to σ^G^.

### The *sigG* promoter may be aberrantly activated by σ^F^ at late times in the absence of σ^G^

**Fig 3:**
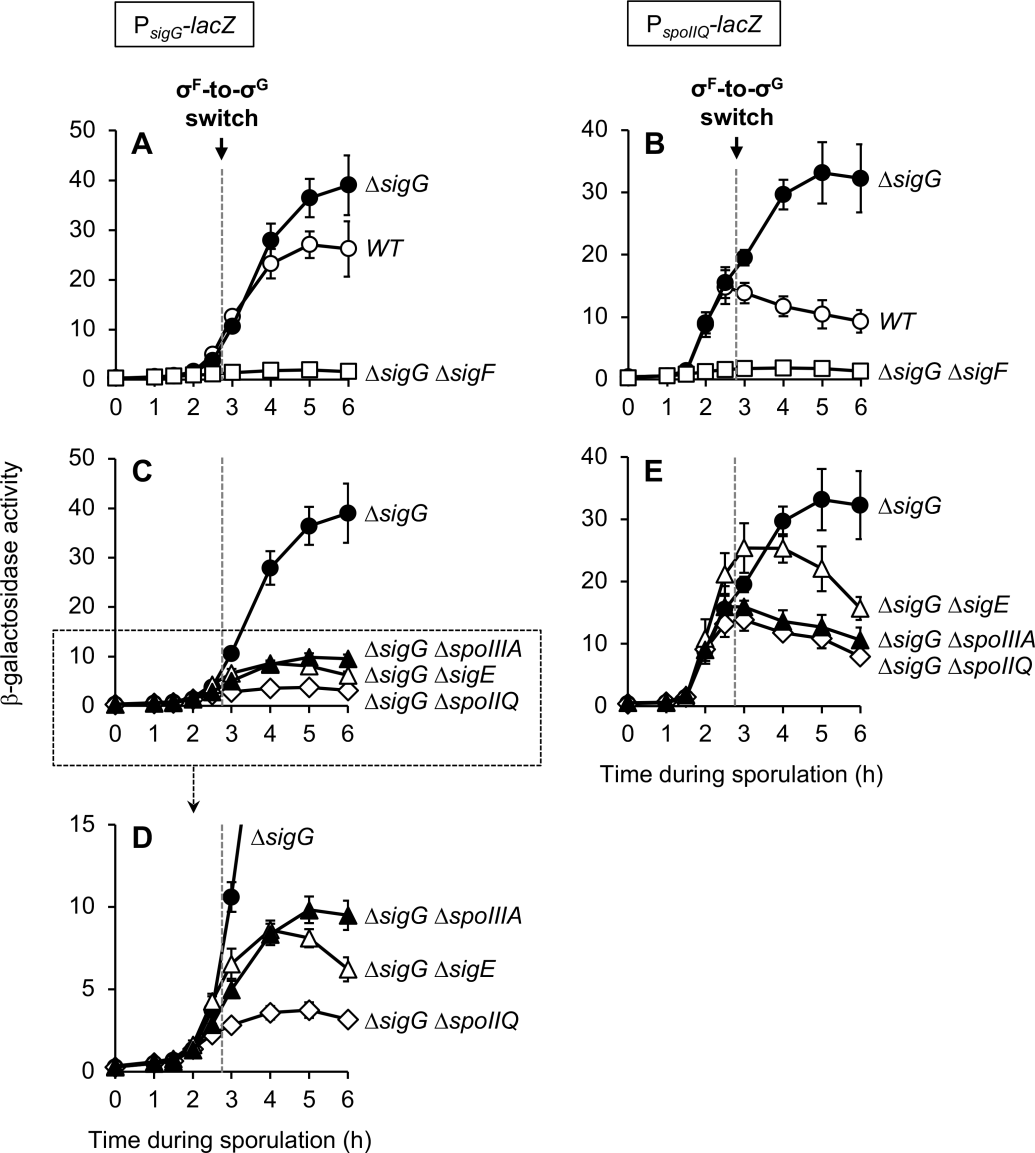
Late σ^F^-dependent expression of both P_*sigG*_ and P_*spoIIQ*_ requires SpoIIQ, σ^E^, and SpoIIIAA-AH. The activity of P_*sigG*_*-lacZ* **(A, C, D)** or P_*spoIIQ*_*-lacZ* **(B, E)** was monitored during sporulation of strains with the following genotypes: wild type (*WT*; open circles), *ΔsigG* (closed circles), *ΔsigG ΔsigF* (open squares), *ΔsigG ΔspoIIQ* (open diamonds), *ΔsigG ΔsigE* (open triangles), and *ΔsigG ΔspoIIIAA-AH* (closed triangles). (P_*sigG*_*-lacZ* strains were JJB31, JJB73, JJB75, JJB79, JJB85, and JJB77, respectively. P_*spoIIQ*_*-lacZ* strains were AHB881, AHB882, AHB915, AHB916, AHB917, and AHB1017, respectively.) For clarity, only data from a subset of these strains are presented in each graph (as labeled) and, also for clarity, the data for the *ΔsigG* strain of each (closed circles) is presented in all graphs. Note that (D) provides a zoomed view of the data from the boxed area of (C). The timing of the σ^F^-to-σ^G^ switch, between sporulation hours 2.5 and 3, is indicated in each panel by a dashed gray line. For all panels, error bars indicate ± standard deviations based on three independent experiments.

In the generally accepted model for regulation of *sigG* expression, *sigG* is transcribed first under the control of σ^F^ (albeit with unique regulation, see Introduction and below) and then under the control of σ^G^ in an auto-regulatory loop. To determine the contribution of σ^F^ versus σ^G^ to the transcription of P_*sigG*_ (using our strongest reporter, P_*sigG*_*-ATG-comGA*^*2-8*^*-lacZ*, henceforth simply P_*sigG*_*-lacZ*), we deleted the gene encoding σ^G^ (*sigG*) alone or in combination with *sigF*, which encodes σ^F^. We predicted that *ΔsigG* would eliminate any late P_*sigG*_ activity that depends upon σ^G^, leaving intact the σ^F^-dependent contribution to P_*sigG*_ expression, which would then be eliminated upon introduction of *ΔsigF*. However, we found that P_*sigG*_*-lacZ* expression was not at all reduced at any time by *ΔsigG*, but in fact was slightly stimulated at later times; yet as predicted, the *ΔsigF ΔsigG* double mutant displayed no detectable β-galactosidase activity at any timepoint (Fig 3A). Close examination of the literature revealed that this effect of *ΔsigG* upon P_*sigG*_ should not have been unexpected. Some of the earliest studies of *sigG* (then called *spoIIIG*) reported that P_*sigG*_ activity was unaltered or even stimulated by *sigG* mutation [9,34,35], although other studies observed modest or even significant decreases [20,36].

One interpretation of our results could be that all P_*sigG*_ activity, at both early and late times during sporulation, is due exclusively to σ^F^ and not at all to σ^G^. However, we find this to be an unsatisfactory interpretation given that σ^G^ has been shown to activate P_*sigG*_ *in vitro* and in directed *in vivo* experiments [9,37,38]. Furthermore, there is no evidence that σ^F^ remains active at later times of sporulation during which σ^G^ is active; in fact, in one study, σ^F^ and σ^G^ activities could not be detected to overlap [5].

We instead suggest an alternative explanation for these data, namely that the late expression of P_*sigG*_*-lacZ* in *ΔsigG* cells is due to *aberrant* σ^F^ activity that is known to be unmasked in the absence of the late-acting sigma factor σ^G^ [13,15,39]. The cause of this aberrant activity is poorly understood but likely involves the σ^F^ inhibitor Fin, which is expressed partly under the control of σ^G^, as well as other σ^G^-dependent, Fin-independent mechanisms [10,11]. To demonstrate the likelihood that P_*sigG*_*-lacZ* is aberrantly activated by σ^F^ at late times in *ΔsigG* cells, we repeated the experiment presented for P_*sigG*_*-lacZ* (Fig 3A), but with strains harboring the exclusively σ^F^-dependent P_*spoIIQ*_*-lacZ* reporter gene. As shown in Fig 3B and as we have previously reported [13], deletion of *sigG* unmasks a late phase of P_*spoIIQ*_*-lacZ* expression from hours 3–5 of sporulation. All P_*spoIIQ*_*-lacZ* expression was eliminated by the further introduction of *ΔsigF* (Fig 3B), indicating that the early (normal) and late (abnormal) transcription of P_*spoIIQ*_*-lacZ* in *ΔsigG* cells is driven by σ^F^. It therefore seems plausible that the *sigG* promoter, like the *spoIIQ* promoter, is subject to aberrant late transcription by σ^F^ in the absence of σ^G^. Importantly, this could make it appear as if σ^F^—and not σ^G^—ordinarily drives P_*sigG*_ transcription at later times during sporulation. As such, we conclude that the contribution of σ^F^ versus σ^G^ to the transcription of P_*sigG*_ in wild type cells cannot be determined with confidence through the use of a *ΔsigG* mutant.

### P_*sigG*_ is unlikely to be the target of a specific intercellular signaling pathway

Despite our inability to genetically dissect the contribution of σ^F^ versus σ^G^ to the activation of our P_*sigG*_*-lacZ* reporter, we reasoned that we could tentatively assign its early expression (hours 2–2.5) to σ^F^ and its late expression (hours 3–6) to σ^G^, based on the timing of the switch from σ^F^ to σ^G^ under our conditions (between hours 2.5 and 3, Fig 1B). This simple interpretation is complicated, however, by the possibility that P_*sigG*_ may not be a typical σ^F^-dependent promoter. Unlike other σ^F^-target promoters, σ^F^-dependent transcription of P_*sigG*_ has been reported to be delayed and to depend upon the mother cell sigma factor σ^E^ and the forespore protein SpoIIQ (itself under the control of σ^F^) [20–23]. These data have been interpreted as evidence for an intercellular signaling pathway/checkpoint mechanism that specifically delays P_*sigG*_ transcription by σ^F^ [2,24]. Upon closer examination of the original studies, however, we noted that the relevant experiments were performed in strains deleted for *sigG* to eliminate σ^G^-dependent P_*sigG*_ transcription. In light of our finding that P_*sigG*_ may behave aberrantly in the absence of σ^G^, as well as recent strides in our understanding of the function of SpoIIQ (see below), we reasoned that these data and their interpretation warranted re-evaluation.

To begin, we sought to reproduce these original findings with our P_*sigG*_*-lacZ* reporter. As shown in Fig 3C, P_*sigG*_*-lacZ* expression in *ΔsigG* cells was indeed significantly reduced at sporulation hour 3 and later when *spoIIQ* or *sigE* (the gene encoding σ^E^) were deleted. Interestingly, however, β-galactosidase production at earlier times (hours 2–2.5), albeit relatively weak, was unaffected by deletion of *spoIIQ* or *sigE* (Fig 3D). These data indicate that in *ΔsigG* cells, σ^F^-dependent expression of P_*sigG*_ at late times (but not early times) requires σ^E^ and SpoIIQ, a finding that is mostly consistent with previous studies [20,22,23].

In the years since these previous studies were performed, SpoIIQ has been determined to assemble with the eight mother cell proteins SpoIIIAA-AH into a channel apparatus that connects the forespore and mother cell at intermediate stages of sporulation (reviewed in [12]). This SpoIIIAA-AH•SpoIIQ channel is generally required for any late gene expression in the forespore, including that normally directed by σ^G^, abnormally directed by σ^F^ (as in a *ΔsigG* mutant), or engineered to be directed by a heterologous RNAP [13]. We reasoned, therefore, that the σ^E^- and SpoIIQ-dependence of late σ^F^-dependent P_*sigG*_ expression might simply be another example of late forespore gene expression requiring the SpoIIIAA-AH•SpoIIQ channel (note that the *spoIIIAA-AH* operon is expressed under σ^E^ control). To investigate this possibility, we tested whether late P_*sigG*_ activity in *ΔsigG* cells also depended upon the channel proteins SpoIIIAA-AH. As shown in Fig 3C, introduction of *ΔspoIIIAA-AH* caused P_*sigG*_*-lacZ* expression to be significantly reduced at late times (hour 3 and later) in a manner that was comparable to that observed with *ΔsigE* and *ΔspoIIQ*. And like *ΔsigE* and *ΔspoIIQ*, *ΔspoIIIAA-AH* did not alter β-galactosidase production at early times (hours 2–2.5) (Fig 3D). These findings therefore indicate that P_*sigG*_ expression is dependent at late (but not early) times upon SpoIIQ, σ^E^, and SpoIIIAA-AH. Although we cannot exclude the possibility of pleiotropic effects of these deletion mutants, we believe that the simplest interpretation of these data is that P_*sigG*_ expression at late times is dependent—as appears to be any late forespore gene expression—upon the SpoIIIAA-AH•SpoIIQ channel.

To further demonstrate the likelihood that P_*sigG*_ is not subject to unique regulation by SpoIIQ, σ^E^, and SpoIIIAA-AH, we repeated the experiment presented for P_*sigG*_*-lacZ* (Fig 3C and 3D) with strains harboring the σ^F^-dependent P_*spoIIQ*_*-lacZ* reporter gene. As shown in Fig 3E and (in part) as we have previously reported [13], the aberrant, late σ^F^-directed expression of P_*spoIIQ*_*-lacZ* in *ΔsigG* cells was similarly dependent upon *spoIIQ*, *sigE*, and *spoIIIAA-AH*. (The relatively higher residual expression in the *ΔsigE* mutant is likely due to the abnormal formation of two forespore compartments, each with active σ^F^ [40].) Also similar to our findings for P_*sigG*_*-lacZ*, these mutations did not alter P_*spoIIQ*_*-lacZ* expression at early times (hours 2–2.5) (Fig 3E). We therefore conclude that P_*sigG*_ and P_*spoIIQ*_ both require *spoIIQ*, *sigE*, and *spoIIIAA-AH* for σ^F^-dependent expression at late (but not early) times in *ΔsigG* cells. Overall, these findings are consistent with the general dependence of late forespore gene expression upon the SpoIIIAA-AH•SpoIIQ channel and, conversely, cast significant doubt upon the conclusion that these proteins comprise a regulatory pathway that specifically delays *sigG* transcription.

### Suboptimal spacing between the P_*sigG*_ -10 and -35 elements partially accounts for low-level expression of P_*sigG*_

**Fig 4:**
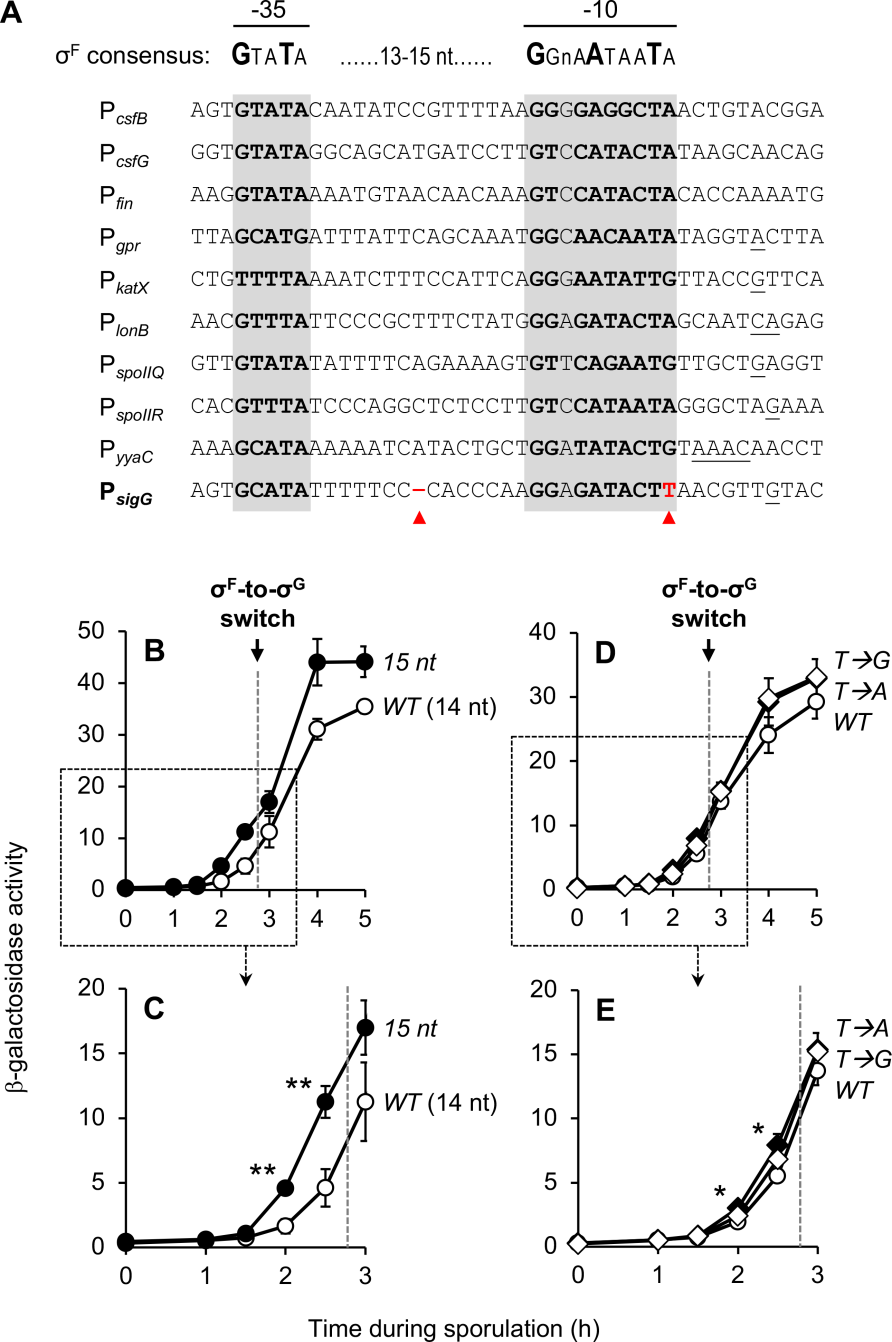
Suboptimal spacing of the P_*sigG*_ -10 and -35 elements diminishes *sigG* expression. **(A)** Alignment of ten *B*. *subtilis* promoters activated by σ^F^, including P_*sigG*_. Nucleotides comprising the -10 and -35 elements of each promoter are in bold and shaded gray. Transcription start sites, if known, are underlined [41]. The consensus promoter sequence for σ^F^ is shown above [38]. Red arrowheads indicate two notable features of P_*sigG*_ that differ from the other σ^F^-target promoters: shorter spacing of the -10 and -35 promoter elements (14 nt as opposed to the more common 15 nt; left arrowhead), and a T at position -7 (more typically an A or G; right arrowhead). **(B, C)** P_*sigG*_ activation is significantly stimulated by increasing the spacing between the -10 and -35 elements to 15 nt. β-Galactosidase production was monitored during sporulation of strains harboring P_*sigG*_*-lacZ* (*WT* [14 nt]; open circles) and ^*15nt*^P_*sigG*_*-lacZ* (*15 nt*; closed circles), a variant in which a single nucleotide was inserted between the P_*sigG*_ -10 and -35 elements to increase their spacing to 15 nt. (Strains JJB31 and JJB51, respectively.) Note that (C) provides a zoomed view of the data from the boxed area of (B). **(D, E)** The T at position -7 at most only modestly influences P_*sigG*_ activation. The activity of P_*sigG*_*-lacZ* (*WT*; closed circles),^*T→A*^P_*sigG*_*-lacZ* (*T→A*; closed diamonds), and ^*T→G*^P_*sigG*_*-lacZ* (*T→G*; open diamonds) was measured during sporulation. (Strains JJB31, JJB87, and JJB89, respectively.) The *T→A* and *T→G* variants were engineered to harbor an A or G, respectively, at position -7 in place of T. Note that (E) provides a zoomed view of the data from the boxed area of (D). The timing of the σ^F^-to-σ^G^ switch, between sporulation hours 2.5 and 3, is indicated in each panel by a dashed gray line. For all relevant panels, error bars indicate ± standard deviations based on three independent experiments. *p < 0.05, **p < 0.01 Student’s *t*-test.

If P_*sigG*_ transcription is not delayed by an intercellular regulatory pathway, then why is *sigG* not more robustly expressed at early times? Bioinformatically the -10 and -35 elements of P_*sigG*_ are recognized as a good match to the σ^F^ consensus (Figs 1D and 4A) [41], and P_*sigG*_ is readily transcribed by σ^F^•RNAP *in vitro* [9]. The *sigG* RBS and its spacing from the start codon (Fig 1D) appear to be ideal for translation initiation [30]. And, as we have shown, the less common GTG start codon of *sigG*, as well as its native 5’ coding sequence, only modestly reduce its translation efficiency (Fig 2B and 2D). We therefore reasoned that P_*sigG*_ may be subject to a currently unknown, additional mode of negative regulation *in vivo* that weakens its expression at early times and, perhaps in turn, helps to properly time the switch to late, σ^G^-directed gene expression in the developing spore.

To identify novel mechanisms of *sigG* regulation, we first turned our attention to the P_*sigG*_ -10 and -35 promoter elements. As shown in Fig 4A, there were two notable differences when we compared P_*sigG*_ to several other promoters also recognized by σ^F^ in *B*. *subtilis*. First was that the spacing between the P_*sigG*_ -10 and -35 elements is 14 nts, whereas the majority of promoters had a spacing of 15 nts. Second, the nucleotide at position -7 in P_*sigG*_ is a T, whereas in the majority of promoters the equivalent position is an A or G.

To test whether one or both of these unique features could explain the low level expression of P_*sigG*_, we constructed variants of our P_*sigG*_*-lacZ* reporter in which the spacing between the -10 and -35 elements was increased to 15 nt by insertion of single base pair (^*15nt*^P_*sigG*_*-lacZ*), or in which the T at position -7 was switched to A or G (^*T→A*^P_*sigG*_*-lacZ* or ^*T→G*^P_*sigG*_*-lacZ*, respectively). As shown in Fig 4B, the ^*15nt*^P_*sigG*_*-lacZ* reporter displayed a notable increase in expression relative to the corresponding wild type reporter at both early and late times of sporulation. Interestingly, this stimulation was most pronounced at times corresponding to σ^F^ activity: at hours 2 and 2.5, the ^*15nt*^P_*sigG*_*-lacZ* reporter displayed a nearly 3-fold increase in expression (Fig 4C). In contrast, switching the T at position -7 to either A or G had very little effect on the extent of P_*sigG*_ expression, although a slight, statistically-significant increase for the ^*T→A*^P_*sigG*_*-lacZ* reporter (~1.5-fold relative to the wild type P_*sigG*_*-lacZ* reporter) was detected at hours 2 and 2.5 (Fig 4D and 4E). Finally, we combined the *15nt* alteration with the *T→A* or *T→G* mutations to test for a synergistic effect on P_*sigG*_ activity, but no additional stimulation was observed (S2 Fig). Together these findings suggest that the ability of σ^F^ to activate P_*sigG*_ at early times is considerably diminished by the shorter spacing between the P_*sigG*_ -10 and -35 elements, and is only modestly (if at all) affected by the identity of the nucleotide at position -7 (T vs. A vs. G).

### *sigG* translation is reduced by formation of an mRNA stem-loop structure that occludes the *sigG* RBS

**Fig 5:**
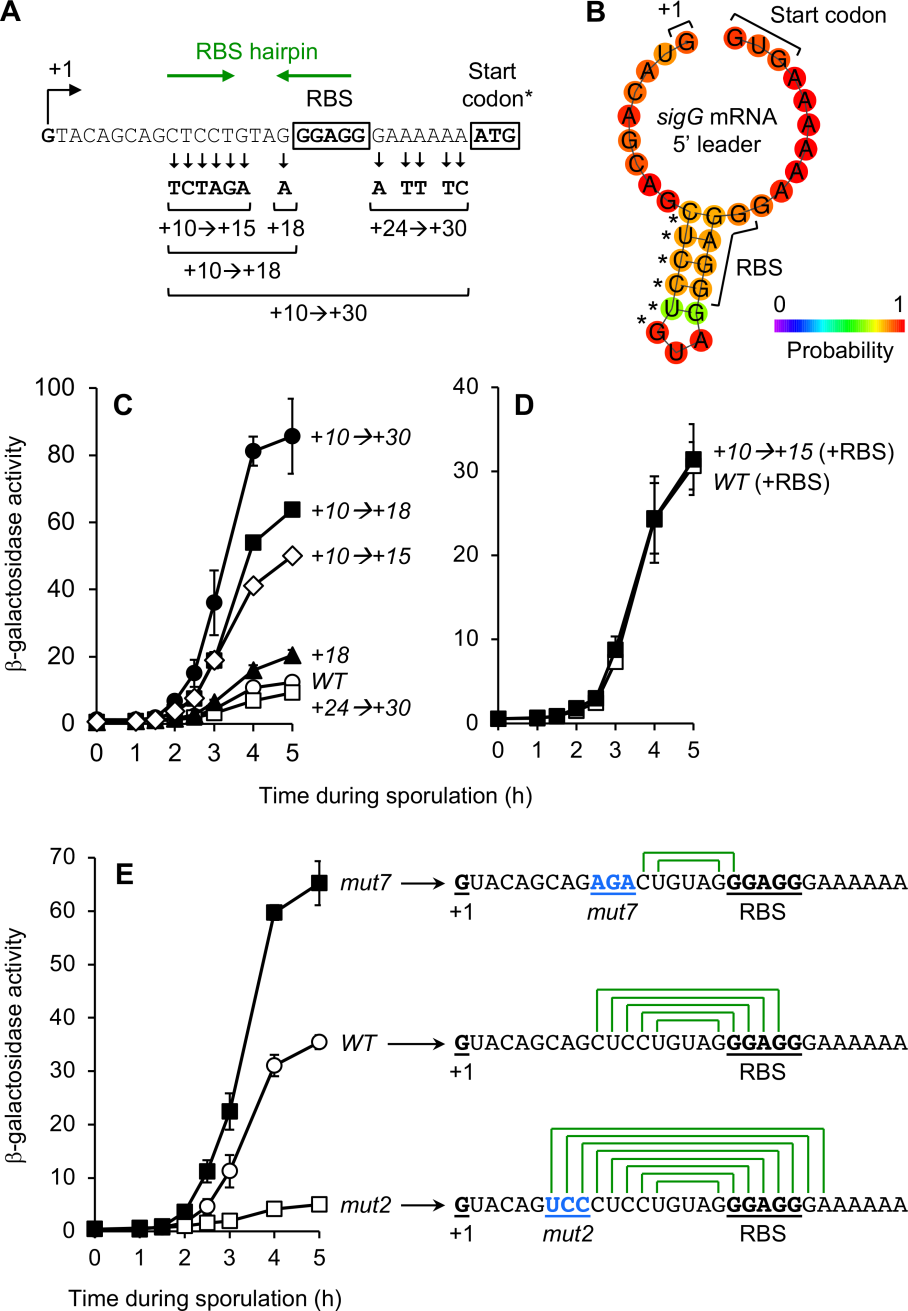
An mRNA hairpin formed between the *sigG* leader sequence and RBS significantly decreases P_*sigG*_*-lacZ* translation. **(A)** Depiction of the mutations in the P_*sigG*_^*+10→+30*^*-lacZ* reporter variant, as well as variants designed to harbor only a subset of these mutations. The *sigG* transcription start site (+1), *sigG* ribosome-binding site (RBS), and ATG start codon are indicated. (*Note that the P_*sigG*_*-lacZ* reporter gene used here harbored the non-native ATG start codon in place of the native *sigG* GTG start codon.) Also indicated, with green arrows, are complementary sequences predicted to form the RBS hairpin structure shown in (B). **(B)** Depiction of the *sigG* RBS-hairpin structure predicted to form in the *sigG* mRNA leader sequence. The 5’ end of the *sigG* mRNA (+1), the *sigG* core RBS, and native GTG (GUG) translation start codon are labeled. Asterisks indicate the six nucleotide positions altered in the +10*→*+15 mutant. The prediction and graphic were generated by ViennaRNA, with bases color-coded according to their partition function probabilities [68]. **(C)** Alteration to nucleotides upstream of the *sigG* RBS stimulates expression from a P_*sigG*_*-lacZ* reporter gene. The activities of P_*sigG*_^*+10→+30*^*-lacZ* (*+10→+30*; closed circles), P_*sigG*_^*+24→+30*^*-lacZ* (*+24→+30*; open squares), P_*sigG*_^*+10→+18*^*-lacZ* (*+10→+18*; closed squares), P_*sigG*_^*+18*^*-lacZ* (*+18*; closed triangles), P_*sigG*_^*+10→+15*^*-lacZ* (*+10→+15*; open diamonds), and the corresponding wild type P_*sigG*_*-lacZ* (*WT*; open circles) were measured during sporulation. (Strains AHB883, AHB2124, AHB2126, JC68, JC70, and AHB1274, respectively.) **(D)** The *+10→+15* alterations do not impact β-galactosidase production from a transcriptional *lacZ* reporter. β-Galactosidase production was monitored during sporulation of strains harboring P_*sigG*_*-RBS-lacZ* (*WT* [+RBS]; open squares) and P_*sigG*_^*+10→+15*^*-RBS-lacZ* (*+10→+15* [+RBS]; closed squares). (Strains AM3 and AM4, respectively.) In these transcriptional reporters, *lacZ* is separated from the *sigG* RBS by a spacer and provided with an engineered RBS. **(E)** Mutations that weaken/eliminate or strengthen the *sigG* RBS-hairpin increase or decrease P_*sigG*_*-lacZ* expression, respectively. The activities of P_*sigG*_^*mut7*^*-lacZ* (*mut7*; closed squares), P_*sigG*_^*mut2*^*-lacZ* (*mut2*; open squares), and the corresponding wild type P_*sigG*_*-lacZ* (*WT*; open circles) were measured during sporulation. (Strains JJB55, JJB37, and JJB31, respectively.) The mRNA leader sequence for each P_*sigG*_*-lacZ* variant is indicated to the right, with the 5’ end of the mRNA (+1), core RBS, and the *mut7* or *mut2* mutations (in blue) labeled and underlined. Also depicted is the capacity for formation of a hairpin structure (green lines connecting complementary base-pairs). For all relevant panels, error bars indicate ± standard deviations based on three independent experiments.

Next, our attention was drawn to the possibility that nucleotides adjacent to the *sigG* RBS might also contribute to the low levels of *sigG* expression. This idea came from characterization of a P_*sigG*_*-lacZ* variant, originally constructed for another line of investigation, in which 12 nucleotides ranging from positions +10 to +30, both upstream and downstream of the *sigG* core RBS, were randomly mutated (Fig 5A). (Note that the P_*sigG*_*-lacZ* reporter utilized here also harbored an ATG start codon in place of the native GTG start codon.) As shown in Fig 5C, this P_*sigG*_^*+10→+30*^*-lacZ* reporter was expressed ~5-8-times more robustly than the corresponding wild type reporter at both early and late times of sporulation. To identify the nucleotides responsible for this effect, we constructed P_*sigG*_*-lacZ* variants harboring subsets of the original 12 mutations (Fig 5A). Through this analysis, we discovered that the 6 alterations introduced at positions +10 through +15 accounted for the majority of the observed stimulation. β-Galactosidase production from P_*sigG*_^*+10→+15*^*-lacZ* was increased ~3-4-fold at both early and late times of sporulation compared to the corresponding wild type P_*sigG*_*-lacZ*, while all other mutations either had no effect or only modestly affected expression (Fig 5C).

Positions +10 through +15 are located downstream of the *sigG* transcription start site (+1) and upstream of the core *sigG* RBS (positions +19 to +23) (Fig 5A). These nucleotides therefore might exert their inhibitory effect upon *sigG* expression at the level of transcription, mRNA stability, and/or mRNA translation. To distinguish these modes of regulation, we introduced the *+10→+15* mutations into a transcriptional P_*sigG*_*-lacZ* reporter (referred to here as P_*sigG*_*-RBS-lacZ*), in which *lacZ* was separated from the *sigG* RBS by a spacer and provided with an engineered, optimal RBS. As shown in Fig 5D, β-galactosidase production from the P_*sigG*_*-RBS-lacZ* reporter was unaffected by the *+10→+15* mutations, arguing strongly for a role of these nucleotides specifically in the regulation of translation initiation at the *sigG* RBS.

One mechanism for regulation of translation initiation involves the occlusion of the RBS by complementary base-pairing with adjacent nucleotides in the 5’ mRNA leader sequence (reviewed in [42]). To determine whether this may be the case for *sigG*, we analyzed the *sigG* 5’ mRNA leader sequence for potential secondary structures. Strikingly, we found that a stem-loop structure comprised of five sequential base-pairs was predicted to form between the majority of the *sigG* RBS and upstream nucleotides (Fig 5A and 5B), with a calculated free energy of -6.2 kcal/mol [43]. Interestingly (and serendipitously), five of the six nucleotides altered in the *+10→+15* mutant correspond exactly to the five nucleotides that participate in formation of this *sigG* “RBS hairpin” (asterisks in Fig 5B). Re-analysis of the *sigG*^*+10→+15*^ 5’ mRNA leader sequence for potential secondary structure confirmed that this mutant was no longer predicted to form this hairpin [43]. Together, these findings are suggestive of a model in which *sigG* translation is ordinarily dampened by a stem-loop structure that occludes the *sigG* RBS.

To further confirm the role of the identified RBS hairpin in regulating *sigG* translation, we introduced mutations into our P_*sigG*_*-lacZ* reporter that were specifically designed to weaken/eliminate (“*mut7*”) or strengthen (“*mut2*”) the *sigG* RBS hairpin (Fig 5E, right). As expected, the P_*sigG*_^*mut7*^*-lacZ* reporter, which retained the potential for only two complementary base pairs, was expressed more robustly, producing ~2-fold more β-galactosidase than the corresponding wild type reporter at both early and late times of sporulation (Fig 5E). We do note that this effect was not as robust as the 3-4-fold stimulation we observed for the *+10→+15* variant (see Fig 5C). Although we cannot be certain, we speculate that this difference is not due to residual hairpin formation, but rather secondary consequences of these mutations (such as decreased mRNA stability). In contrast, the “strengthened hairpin” P_*sigG*_^*mut2*^*-lacZ* reporter displayed very low levels of expression (Fig 5E). Altogether, these findings indicate that the *sigG* RBS hairpin ordinarily dampens *sigG* translation by 2-4-fold and, when strengthened, can almost entirely block translation from the *sigG* mRNA.

### Together, the identified transcriptional and translational regulation of *sigG* diminishes expression by 4-6-fold

**Fig 6:**
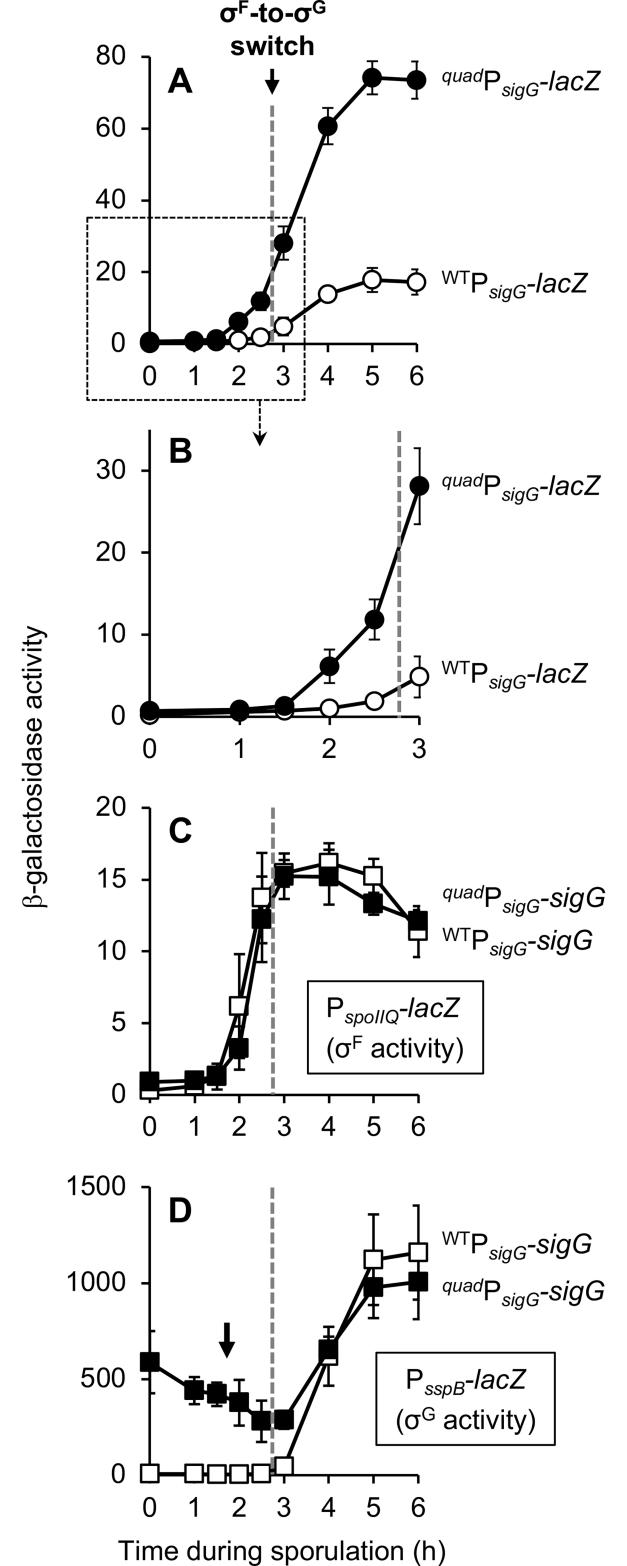
The identified transcriptional and translational regulation of *sigG* diminishes *sigG* expression by 4-6-fold and is required to prevent aberrant activity of σ^G^. **(A, B)** Expression of a P_*sigG*_ reporter is increased by 4-6-fold in the absence of the four regulatory features identified in this study. β-Galactosidase production was monitored during sporulation of strains harboring P_*sigG*_*-sigG*^*1-28*^*-lacZ* (^WT^P_*sigG*_*-lacZ*; open circles) or a variant in which all four features of *sigG* that dampen expression were simultaneously removed or repaired, ^*15nt*^P_*sigG*_^*mut7*^*-ATG-*^*RSS*^*sigG*^*2-28*^*-lacZ* (^*quad*^P_*sigG*_-*lacZ*; closed circles) (strains EBM177 and EBM262, respectively.) Note that (B) provides a zoomed view of the data from the boxed area of (A). **(C, D)** Expression of *sigG* from regulatory sequences lacking the four regulatory features identified in this study causes aberrant σ^G^ activity during a time course of sporulation. β-Galactosidase production from (C) the σ^F^-dependent P_*spoIIQ*_*-lacZ* reporter or (D) the σ^G^-dependent P_*sspB*_*-lacZ* reporter was monitored during sporulation of strains in which *sigG* was expressed from its wild type regulatory sequences (^WT^P_*sigG*_*-sigG*; open squares) or from regulatory sequences modified to remove or repair the four features identified in this study to dampen *sigG* expression (^*quad*^P_*sigG*_*-sigG*; closed squares). (P_*spoIIQ*_*-lacZ* strains were CFB429 and CFB431, respectively. P_*sspB*_*-lacZ* strains were CFB435 and CFB437, respectively.) The black arrow in (D) indicates aberrant σ^G^ activity at early times of sporulation. The timing of the σ^F^-to-σ^G^ switch, between sporulation hours 2.5 and 3, is indicated in each panel by a dashed gray line. For all panels, error bars indicate ± standard deviations based on three independent experiments.

Our data reveal that expression of *sigG* is diminished by at least four mechanisms: (i) suboptimal spacing of the P_*sigG*_ -10 and -35 elements, (ii) a hairpin in the *sigG* mRNA 5’ leader sequence predicted to occlude the RBS, (iii) a suboptimal GTG start codon, and (iv) secondary structure in the *sigG* 5’ coding sequence. To visualize the full extent of *sigG* inhibition by these mechanisms, we built a P_*sigG*_*-lacZ* reporter construct (referred to as ^*quad*^P_*sigG*_*-lacZ*) that simultaneously removed or “repaired” all of these features. Strikingly, ^*quad*^P_*sigG*_*-lacZ* was expressed ~4-6-fold more robustly than the original P_*sigG*_*-sigG*^*1-28*^*-lacZ* reporter (Fig 6A and 6B). This effect is in line with what would be predicted by the individual effects of each alteration (4-13-fold), suggesting that these four features operate independently to modulate *sigG* expression during sporulation. Importantly, this 4-6-fold increase is almost certainly an underestimate of the actual increase that would result if the *sigG* gene itself (as opposed to a *lacZ* reporter gene) were misexpressed, given the amplification of *sigG* expression that would occur via σ^G^ autoregulation.

### Misregulation of *sigG* causes inappropriate σ^G^ activity during vegetative growth and disrupts the timing of σ^G^ activation during sporulation

We hypothesized that the four identified mechanisms of *sigG* negative regulation help to ensure the proper execution of the switch from σ^F^ to σ^G^ in the developing forespore, most likely by preventing premature σ^G^ activation. To test this prediction, we constructed a strain in which *sigG* itself (i.e. not a *lacZ* reporter gene) was expressed from regulatory sequences altered to remove or repair the four features that ordinarily reduce *sigG* expression (these alterations were identical to those in the ^*quad*^P_*sigG*_*-lacZ* reporter, see above). This engineered *sigG* gene, referred to as ^*quad*^P_*sigG*_*-sigG*, was inserted at an ectopic locus in a strain deleted for the native *sigG* gene; a strain harboring *sigG* under the control of wild type regulatory sequences (P_*sigG*_*-sigG*) was constructed in an identical manner as a control. The resulting ^*quad*^P_*sigG*_*-sigG* strain displayed no detectable defect in heat resistant spore formation relative to the wild type P_*sigG*_*-sigG* control strain (S3 Fig), indicating that misregulation of *sigG* does not drastically compromise sporulation.

To determine whether *sigG* misregulation interferes with the switch from σ^F^ to σ^G^, we measured the activities of these two sigma factors during sporulation of the ^*quad*^P_*sigG*_*-sigG* and wild type P_*sigG*_*-sigG* control strains using *lacZ* reporter genes. As shown in Fig 6C, we observed no detectable difference in the timing or extent of σ^F^-dependent β-galactosidase production from a P_*spoIIQ*_*-lacZ* reporter. In contrast, σ^G^-dependent P_*sspB*_*-lacZ* expression was significantly altered such that the ^*quad*^P_*sigG*_*-sigG* strain displayed up to 100-fold more β-galactosidase activity at sporulation hours 0–3, but appeared to be relatively normal thereafter (Fig 6D). These results could be consistent with inappropriate early activation of σ^G^ in the forespores of ^*quad*^P_*sigG*_*-sigG* cells during sporulation, but the presence of significant β-galactosidase activity at hour 0 (i.e. prior to the onset of sporulation) also suggests that σ^G^ may be inappropriately active in vegetative cells.

**Fig 7:**
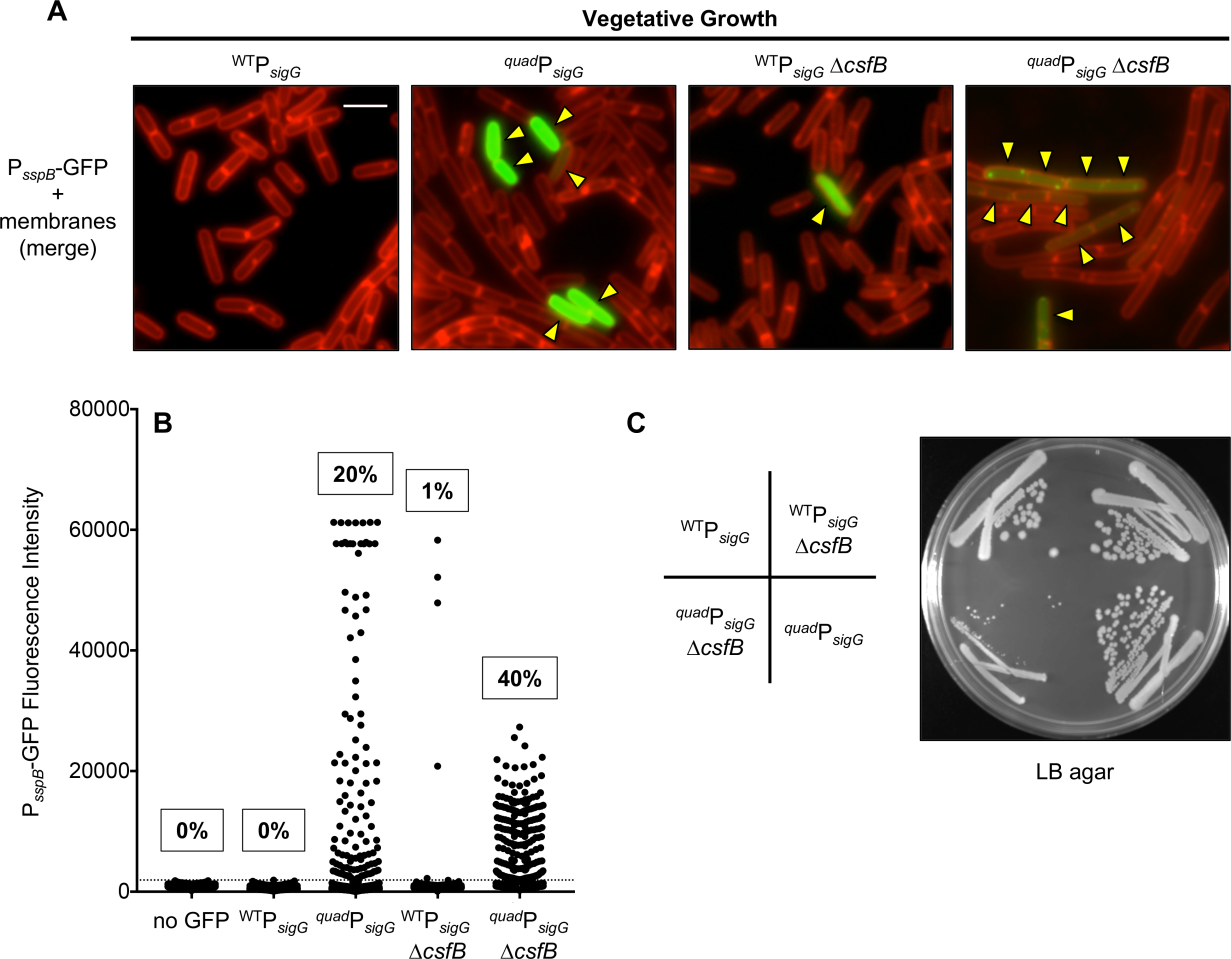
Misexpression of *sigG* causes ectopic σ^G^ activity in a subset of vegetative cells. **(A, B)** GFP production from a σ^G^-dependent P_*sspB*_*-gfp* reporter was monitored by fluorescence microscopy of vegetatively growing cells in which *sigG* was expressed from its wild type regulatory sequences (^WT^P_*sigG*_) or from regulatory sequences modified to remove or repair the four features identified in this study to dampen *sigG* expression (^*quad*^P_*sigG*_). Additionally, cells either harbored wild type *csfB*, or were deleted for the gene (*ΔcsfB*). (Strains EBM192 [^WT^P_*sigG*_], EBM276 [^*quad*^P_*sigG*_], EBM282 [^WT^P_*sigG*_ *ΔcsfB*], and EBM287 [^*quad*^P_*sigG*_-*sigG ΔcsfB*].) In **(A)**, representative microscopy images are shown with GFP fluorescence (false-colored green) merged with membrane fluorescence from the dye FM 4–64 (false-colored red). Yellow arrowheads indicate vegetative cells with visible GFP fluorescence. Scale bar 5 μm. In **(B)**, GFP fluorescence intensity (with background subtracted) for more than 500 cells of each strain, including a “no GFP” control strain (PY79) lacking the P_*sspB*_*-gfp* reporter, is shown in column scatter graph format, with each cell represented by a black dot. Cells exhibiting fluorescence intensity above the cut off value (three standard deviations above mean auto-fluorescence of the “no GFP” strain; gray dashed line) were determined to have detectable σ^G^ activity. The percentage of cells with this activity is indicated above the respective strain. Note that ~60000 fluorescence units is the upper limit of detection under our microscopy settings. **(C)** Simultaneous deletion of *csfB* and misexpression of *sigG* from ^*quad*^P_*sigG*_ causes synthetic toxicity to vegetatively growing *B*. *subtilis*. The same strains described in (A) were struck onto LB agar and were photographed after ~18 hours of growth at 37°C.

To identify the sub-population(s) of ^*quad*^P_*sigG*_*-sigG* cells exhibiting inappropriate σ^G^ activity, we monitored and quantified σ^G^-dependent expression of a P_*sspB*_*-gfp* reporter gene in single cells by fluorescence microscopy during both vegetative growth and sporulation. As shown in Fig 7A and 7B, we detected GFP fluorescence significantly above background in 20% of vegetative ^*quad*^P_*sigG*_*-sigG* cells, as compared to 0% of the wild type P_*sigG*_*-sigG* control cells. Inappropriate σ^G^ activity in a subset of vegetative cells has also been reported for cells lacking the σ^G^ inhibitor CsfB [16,44]. Consistent with these reports, introduction of a *ΔcsfB* deletion into our wild type P_*sigG*_*-sigG* control strain caused 1% of vegetative cells to display σ^G^-dependent GFP expression (Fig 7A and 7B). Interestingly, when the ^*quad*^P_*sigG*_*-sigG* and *ΔcsfB* mutations were combined, the resulting double mutant exhibited aberrant σ^G^ activity in 40% of vegetative cells (Fig 7A and 7B). The double mutant also appeared sickly: the intensity of GFP fluorescence per cell was lower than in the respective single mutants (Fig 7A and 7B), and GFP protein aggregates accumulated in most cells (Fig 7A). Last, it is worth noting that the ^*quad*^P_*sigG*_*-sigG ΔcsfB* double mutant was difficult to construct and gave rise to very small colonies when grown on LB plates, unlike the two single mutants (Fig 7C). Growth curve analysis in liquid LB media revealed that the double mutant had a significantly longer doubling time during log phase (~32 min vs. ~25 min for the corresponding wild type strain), and failed to reach the same maximal cell density during stationary phase (S4 Fig). In contrast, growth of the two single mutants was indistinguishable from wild type with the exception of the ^*quad*^P_*sigG*_*-sigG* strain, which had a slight but statistically significant increase in doubling time during exponential growth (S4 Fig). Together, these data indicate that the *sigG* regulatory features identified in this study act synergistically with CsfB to prevent inappropriate σ^G^ activity during vegetative growth and that, in the absence of this regulation, vegetative cells are at a severe fitness disadvantage during both exponential growth and stationary phase.

**Fig 8:**
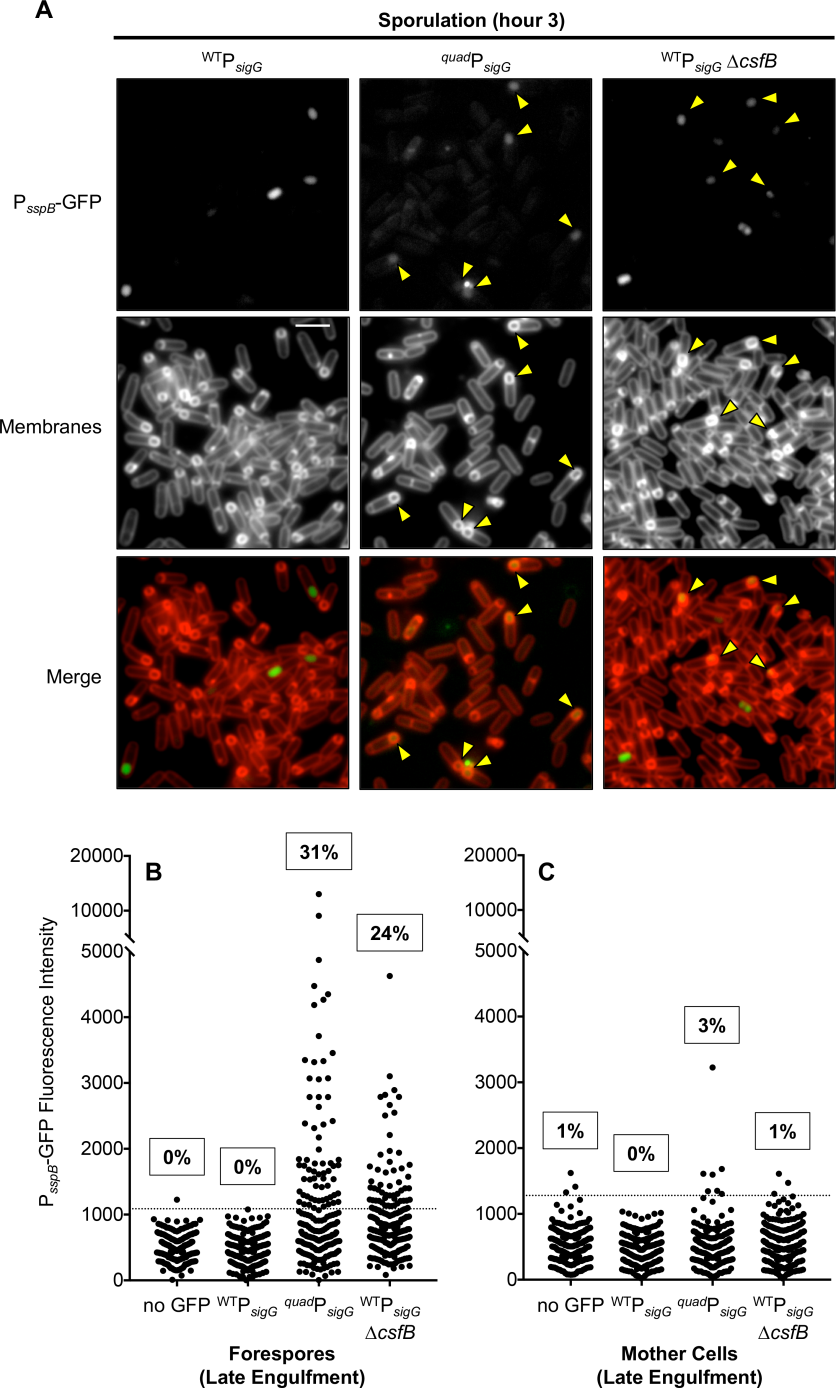
Misexpression of *sigG* causes premature σ^G^ activity in the forespore during sporulation. GFP production from a σ^G^-dependent P_*sspB*_*-gfp* reporter was monitored by fluorescence microscopy of sporulating cells (hour 3.5) in which *sigG* was expressed from its wild type regulatory sequences (^WT^P_*sigG*_) or from regulatory sequences modified to remove or repair the four features identified in this study to dampen *sigG* expression (^*quad*^P_*sigG*_). Additionally, cells either harbored wild type *csfB*, or were deleted for the gene (*ΔcsfB*). (Strains EBM192 [^WT^P_*sigG*_], EBM276 [^*quad*^P_*sigG*_] and EBM282 [^WT^P_*sigG*_ *ΔcsfB*].) **(A)** Representative microscopy images for each strain. GFP fluorescence is depicted in grayscale (P_*sspB*_-GFP) or false-colored green (Merge). Membrane fluorescence from the dye FM 4–64 is shown in grayscale (Membranes) or false-colored red (Merge). Following asymmetric division and during engulfment, the FM 4–64 dye labels all membranes including the double membranes separating the mother cell and forespore; after the completion of engulfment, the membranes surrounding the forespore are no longer accessible to the dye and therefore remain unlabeled. Yellow arrowheads indicate late engulfment forespores, identifiable by their FM 4-64-labeled engulfing membranes, with visible GFP fluorescence. Scale bar 5 μm. **(B)** GFP fluorescence intensity (with background subtracted) for more than 200 late-engulfment forespores or **(C)** their corresponding mother cells for the three strains described in (A) as well as a “no GFP” control strain (PY79) lacking the P_*sspB*_*-gfp* reporter are shown in column scatter graph format, with each cell represented by a black dot. Cells exhibiting fluorescence intensity above the cut off value (three standard deviations above mean auto-fluorescence of the “no GFP” strain; gray dashed line) were determined to have detectable σ^G^ activity. The percentage of cells with this activity is noted above the respective strain.

Next, we observed cells during sporulation to determine whether misexpression of *sigG* might also cause σ^G^ to become prematurely active in the forespore. As expected, σ^G^-dependent expression of the P_*sspB*_*-gfp* reporter gene in wild type P_*sigG*_*-sigG* forespores was observed only after the process of forespore engulfment was complete, and was not detected in any mother cells (Fig 8A–8C). In contrast, when *sigG* expression was misregulated in the ^*quad*^P_*sigG*_*-sigG* mutant, 31% of forespores displayed aberrant σ^G^ activity prior to engulfment (Fig 8A and 8B). Based on a previous report [16], we anticipated that deletion of *csfB* in the wild type P_*sigG*_*-sigG* control strain might give rise to a similar effect. Indeed, we detected significant σ^G^-dependent GFP fluorescence in 24% of *ΔcsfB* forespores that had not yet completed engulfment (Fig 8B). In both cases, aberrant σ^G^ activity was specific to forespores, given that only 3% of ^*quad*^P_*sigG*_*-sigG* mother cells and 1% of *ΔcsfB* mother cells displayed σ^G^-dependent fluorescence (Fig 8C). Unfortunately, due to the poor vegetative growth of the double ^*quad*^P_*sigG*_*-sigG ΔcsfB* mutant (Fig 7C), as well as the pre-existing σ^G^ activity in 40% of vegetative cells (Fig 7A and 7B), we were unable to determine with confidence the combined influence of these mutations on premature σ^G^ activation during sporulation. Nevertheless, our findings reveal that proper regulation of *sigG* expression by the features identified in this study are required to prevent premature σ^G^ activation in the developing forespore.

## Discussion

In this study, we sought to unravel a decades old puzzle in the *B*. *subtilis* sporulation pathway; namely, how does the late acting transcription factor σ^G^ become active at the proper time during forespore development? At the outset, we hypothesized that precise regulation of the transcription and/or translation of *sigG* plays a key role in ensuring that σ^G^ does not become active prematurely. Here we report a careful re-evaluation of *sigG* expression that calls into question long-standing notions regarding its regulation, including models in which *sigG* expression is delayed or dependent upon an intercellular “checkpoint”. Instead, our data support a new working model in which *sigG* expression is neither delayed nor subject to a specific intercellular regulatory pathway, but rather is dampened at the transcriptional and translational levels by at least four features intrinsic to its regulatory and coding sequence. As we hypothesized, these features are required to prevent premature σ^G^ activity in the forespore. We also found that they are required to prevent inappropriate σ^G^ activity in vegetatively growing cells, suggesting that these controls serve not only to fine-tune the timing of σ^G^ activation during sporulation, but also to prevent its inappropriate activation under non-sporulating conditions.

### A new working model for regulation of *sigG* expression

The long-standing working model for regulation of *sigG* expression (see, for example, [2,24]) posits that an intercellular checkpoint mechanism, perhaps involving SpoIIQ, delays σ^F^-dependent *sigG* expression until the early phase of σ^E^-dependent, mother cell gene expression is underway. Here we present evidence that the data supporting this original working model (see Introduction and Results) can be reinterpreted in a manner that does not invoke the existence of an enigmatic, *sigG*-specific intercellular checkpoint. First, the apparently delayed activation of P_*sigG*_ by σ^F^ can be ascribed to aberrant, late σ^F^ activity that is unmasked in the absence of σ^G^ (note that the original experiments were performed in a *ΔsigG* genetic background) [13,15,39]. Second, the dependence upon *sigE* and *spoIIQ* can be explained by the failure of these mutants to assemble the SpoIIIAA-AH•SpoIIQ channel, which is required for any late gene expression in the forespore, including that directed by aberrantly active σ^F^ [13].

As such, we propose here a new, simpler working model for *sigG* regulation (Fig 1C). At early times, we posit that *sigG* is transcribed under the control of σ^F^ without delay, but at levels that are very low and therefore difficult to detect. Then, at later times, σ^G^ drives its own transcription in an autoregulatory loop, during which time the majority of *sigG*/σ^G^ is produced. We have established here that at least four features of the *sigG* regulatory and coding sequences, affecting both transcription and translation, account for its low-level expression: reduced spacing between the -35 and -10 promoter elements, a GTG start codon, a suboptimal 5′ coding sequence, and an RBS-sequestering hairpin located in the 5’ leader sequence.

#### Promoter spacer

The influence of the spacing between the -35 and -10 promoter elements upon promoter activity reflects the requirement that the sigma factor subunit of RNAP simultaneously bind to both sites. Even single base-pair increases or decreases in the spacer length can significantly affect promoter activity (for example, [45–48]). Here, we determined that sub-optimal, reduced spacing between the *sigG* promoter elements does contribute to the weak expression of *sigG*. Interestingly, lengthening the promoter spacer most significantly increased expression during early sporulation, perhaps suggesting that σ^F^ is more sensitive to promoter element spacing than σ^G^. Furthermore, this transcriptional mechanism for dampening *sigG* expression appears to be conserved among endospore formers from the class *Bacilli*. In a survey of 15 representative species from this class (including *B*. *subtilis*), we found that 14 display a reduced (14 bp) spacing between their *sigG* promoter elements (S5 Fig). In contrast, endospore formers from the class *Clostridia* more often space their *sigG* promoter elements by 15 bp; only 3 out of 13 species from this class display the reduced 14 bp spacing (S5 Fig). These observations suggest that the spacing between the *sigG* promoter elements is important in the regulation and possibly optimization of the sporulation program in many *Bacilli* species.

#### GTG start codon

In *B. subtilis*, the start codon GTG is weak in terms of translational efficiency compared to TTG and ATG, which have been reported in some cases to produce up to 3- and 7-fold more translational product, respectively [30]. We found that replacing the *sigG* GTG start codon with ATG improved translational efficiency modestly (~1.3-fold). Nevertheless, it is interesting to note that 11 out of 15 *sigG* genes from representative *Bacilli* utilize a non-ATG start codon (6 GTG, 5 TTG). In contrast, only 1 out of 13 representative *Clostridia* utilize a non-ATG start codon (GTG) (S5 Fig). This suggests that, despite the modest impact on translational efficiency, initiating *sigG* translation with non-ATG start codons is advantageous for members of the *Bacilli*.

#### 5’ coding sequence

In addition to the identity of the start codon, the 5’ coding sequence of a gene can also influence translational efficiency, in some cases by orders of magnitude (see for example [33,49–51]). This influence is thought to be mediated by the presence (or lack thereof) of RNA secondary structures and/or rare codons that impede translation initiation (reviewed in [31]). When we replaced the native *sigG* 5’ coding sequence with synonymous codons selected to minimize secondary structure, or with the 5’ coding sequence of the highly expressed gene *comGA*, expression increased by ~1.5- or 2-fold, respectively. We interpret this data to indicate that the *sigG* 5’ coding sequence ordinarily dampens translation efficiency, at least in part through the formation of secondary structures such as those noted in Fig 1D. However, we cannot exclude the possibility that one or more rare codons and/or the abundance of certain tRNAs in the forespore during sporulation, contribute to this effect.

#### RBS hairpin

Finally, we found that *sigG* expression is dampened ~2-4-fold via a hairpin structure that forms between the RBS and nucleotides in the adjacent 5’ mRNA leader sequence. This small “RBS hairpin”, which is comprised of five complementary base pairs, is distinct from the very large hairpin (comprised of 20+ complementary base pairs) proposed to block translation of a read-through transcript originating from the upstream *spoIIGA-sigE* operon (Fig 1D) [9,52]. (This read-through transcript is not required for *sigG* expression during sporulation, nor does the large hairpin form in the transcript originating from the *sigG* promoter [9,52].) Through targeted mutagenesis we were able to weaken/eliminate or strengthen the RBS hairpin with predictable consequences for *sigG* expression. Interestingly, RBS-sequestering hairpins of similar free energies (ranging from -6.0 to -8.9 kcal/mol) are predicted to form in the *sigG* leader sequences of many endospore-forming *Bacilli* species (found in all 15 of the representatives listed in S5 Fig), but are less common or weaker in *Clostridia* (hairpins with free energies of -6.0 kcal/mol or lower were only found in 4 of the 13 representatives listed in S5 Fig) [43]. A subset of these predicted *sigG* RBS-sequestering hairpins are shown in S6 Fig.

In the simplest scenario, the RBS hairpin dampens translation of the *sigG* transcript at all times, with the ribosome gaining access only during transient moments of hairpin unzipping. This is consistent with the fact that weakening and strengthening the hairpin increases and decreases expression, respectively, both before and after the σ^F^-to-σ^G^ switch. However, this doesn’t exclude a second possible scenario in which the *sigG* RBS hairpin changes conformation dynamically during sporulation, such that the RBS is exposed only at later stages of sporulation (i.e. after the switch from σ^F^ to σ^G^). This regulation could occur through the binding of a small molecule, protein, or small RNA (reviewed in [42]). Given the hypothesis that the forespore becomes dependent on nutrients from the mother cell, delivered through the SpoIIIAA-AH•SpoIIQ channel at late times [13], it is tempting to speculate that dynamic control of *sigG* translation could be tuned to a specific metabolite through a riboswitch-like mechanism. However, the *sigG* 5’ leader is relatively short (30 nt) and does not match any known class of riboswitch according to several web-based bioinformatics tools [53–55]. Nevertheless, exploring the possibility that the *sigG* RBS hairpin is regulated, perhaps by a novel mechanism, is an exciting challenge for future work.

### Do *sigG*/σ^G^ regulatory mechanisms serve primarily to protect from σ^G^ toxicity in non-sporulating cells?

A collective take-home message from this and many other studies of *sigG*/σ^G^ is that this gene/protein is subject to layer upon layer of negative regulation, at the transcriptional, translational, and post-translational levels. While it is tempting to imagine that these layers of regulation evolved to ensure the precise timing of σ^G^ activation in the forespore during sporulation, it has become increasingly evident that they function in addition, or in some cases instead, to prevent aberrant σ^G^ activity in the mother cell and/or in non-sporulating, vegetative cells. Given that *sigG* is autoregulated, even very low levels of “leaky” expression could trigger a positive feedback loop of σ^G^ synthesis and activity. The anti-sigma factor SpoIIAB and the serine protease LonA, which inhibit σ^G^ post-translationally, counteract/eliminate σ^G^ produced in the mother cell and in non-sporulating cells, with no apparent role in σ^G^ regulation in the forespore [28,44,56–59]. In contrast, the anti-sigma factor CsfB post-translationally inhibits σ^G^ in the forespore (at early times), mother cell, and in non-sporulating cells, an impressive feat that is accomplished via *csfB* regulatory sequences that permit transcription by σ^F^ (at early times in the forespore), σ^K^ (at late times in the mother cell), and σ^G^ itself (in vegetative cells, but not in the forespore) (this study and [15,16,44,59,60]).

Here we have found that the regulatory features that dampen *sigG* transcription and translation, like the aforementioned σ^G^ inhibitors, are not solely dedicated to preventing premature σ^G^ activity in the forespore. While misexpression of *sigG* indeed caused nearly one third of forespores to display premature σ^G^ activity, thus supporting our original hypothesis, it also led to aberrant σ^G^ activity in up to 20% of vegetative cells. (We found no evidence for any effect in the mother cell.) We do not currently know why the *sigG*/σ^G^ autoregulatory loop was triggered in this particular subpopulation of vegetative cells, but we speculate that it could be due to activation of P_*sigG*_ by σ^F^ (itself produced by leaky expression) or spurious transcription through the *sigG* coding sequence. We do note that read-through transcription from the *spoIIGA-sigE* operon [9,52], which resides upstream of the native *sigG* gene, could not have been a contributing factor given that *sigG* was located at an ectopic locus in our strain background.

Strikingly, misexpression of *sigG* in cells also lacking CsfB led to a synergistic and severe effect on vegetative cells: nearly half of the population displayed aberrant σ^G^ activity and the strain was sickly and exhibited a small colony morphology, consistent with the documented toxicity of σ^G^ to vegetative cells [44,61]. Unfortunately, this toxicity was so severe that we were unable to assess synergistic effects in the forespore during sporulation. These results, along with the fact that early σ^G^ activity in the forespore (i.e. in ^*quad*^P_*sigG*_*-sigG* or *ΔcsfB* cells) does not appear to be detrimental to the sporulation program (this study and [15]), raise the speculative idea that the majority of *sigG*/σ^G^ regulation has evolved primarily to prevent ectopic σ^G^ activity in vegetative cells, and not to precisely regulate the switch from early to late gene expression in the developing spore. In this light, a key challenge for future studies will be to understand how σ^G^ escapes these many layers of inhibition in order to drive late gene expression in the developing spore.

## Materials and methods

### General methods

All *B*. *subtilis* strains were derived from the laboratory strain PY79 [62]. Tables of strains, plasmids, primers, and synthetic DNA fragments used in this study, as well as details of strain and plasmid construction, are provided as supplementary material (S1, S2, S3, S4 Tables, S1 Text). Bacterial strains were propagated in lysogeny broth (LB), either in liquid culture or on solid plates with 1.6% agar. When appropriate, antibiotics were included as follows: chloramphenicol (5 μg/mL), erythromycin plus lincomycin (1 μg/mL and 25 μg/mL, respectively), spectinomycin (100 μg/mL), kanamycin (5 μg/mL), and ampicillin (100 μg/mL). For growth curves, colonies grown overnight on LB agar from a frozen stock were inoculated directly (to minimize selection of suppressors) into 1.5 mL liquid LB in clear 12-well flat-bottom plates. Plates were incubated at 37°C with continuous shaking in a Synergy H1M plate reader (BioTek, Inc.), and optical density at 600 nm (OD_600_) was measured every 5 min. To quantify spore formation, cells were induced to sporulate by nutrient exhaustion in Difco sporulation medium (DSM) [63,64]. After 24 h at 37°C, the number of colony-forming units that survived heat treatment (20 min at 80°C) was calculated. For all other experiments, sporulation was induced by the resuspension method [64,65]. β-Galactosidase activity was measured as previously described [13] and is reported in arbitrary units (rate of substrate conversion normalized to cell density), unless otherwise noted.

### Fluorescence microscopy and quantification of σ^G^ activity in single cells

Cells harboring the σ^G^-dependent P_*sspB*_*-gfp* reporter were collected during mid-log phase to observe vegetative cells, or at hour 3 of sporulation to observe forespores and their corresponding mother cells during late-engulfment. Harvested cells were resuspended in 1x phosphate-buffered saline (PBS) with 1 μg/ml FM 4–64 (Molecular Probes) to stain membranes, and spotted on thin 1% agarose pads with poly-L-lysine (Sigma) treated coverslips. Fluorescence microscopy was performed with a Nikon Eclipse Ti-U inverted microscope fitted with filter sets 49002 (Chroma; excitation 470/40x, dichroic 495Ipxr, and emission 525/50m) and 49017 (Chroma; excitation 560/40x, dichroic 590Ipxr, and emission 590lp) for GFP and FM 4–64 detection, respectively. Images were captured with an ORCA-Flash4.0 digital CMOS camera (Hamamatsu Photonics K.K.) using NIS-Elements Advanced Research software (Nikon Instruments Inc.).

For each strain, GFP fluorescence intensity above background was quantified for more than 500 vegetative cells and more than 200 late-engulfment forespores (defined as cells that were more than halfway engulfed, but not fully engulfed as determined by FM 4–64 membrane staining) and their corresponding mother cells, using the ImageJ software distributed within the Fiji package [66,67]. Cells to be quantified were obtained from two or three biological replicates (experiments carried out on different days). For each replicate sample, at least three fields were selected at random from the same slide and images were acquired with identical settings. Cells with a net GFP fluorescence intensity more than three standard deviations higher than autofluorescence (as determined by images collected in parallel from PY79, the wild type parent strain lacking a *gfp* reporter), were considered to have detectable σ^G^ activity. Percentages were calculated based on the total number of cells or forespores quantified. Representative images had background subtraction applied, were false colored, and overlaid using the aforementioned ImageJ software.

## Supporting information

S1 Fig Predicted secondary structure formation by the *sigG* 5’ coding sequence present in *P_sigG_-ATG-sigG^2-28^-lacZ* before and after optimization. An AUG (ATG) start codon followed by codons 2–28 of *B*. *subtilis sigG* are shown (top sequence), as is a sequence comprised of synonymous codons selected by the mRNA Optimizer tool [32] to minimize secondary structure (bottom sequence). Note that the latter is the sequence present in the P_*sigG*_*-ATG-*^*RSS*^*sigG*^*2-28*^*-lacZ* reporter construct used in Fig 2. Nucleotide differences are indicated in red and blue text. Both sequences were analyzed for secondary structure by the RNAstructure method [43]. Potential secondary structures are displayed above (red) or below (blue) using RNAbow diagrams [69], with base pairs indicated by arcs, the thickness and shading of which is proportional to their probability. The partition function free energy (G) for each is indicated.
(TIF)

S2 Fig P*_sigG_* activity is not synergistically stimulated by substitution of the T at position -7 for A or G and increased spacing of the -35 and -10 promoter elements. β-Galactosidase production was monitored during sporulation of strains harboring P_*sigG*_*-lacZ* (*WT*; open circles), ^*15nt*^P_*sigG*_*-lacZ* (*15 nt*; closed circles), ^*T→A*^P_*sigG*_*-lacZ* (*T→A*; open diamonds), ^*T→G*^P_*sigG*_*-lacZ* (*T→G*; closed diamonds) ^*15nt*,*T→A*^P_*sigG*_*-lacZ* (*15 nt*, *T→A*; open triangles), and ^*15nt*,*T→G*^P_*sigG*_*-lacZ* (*15 nt*, *T→G*; open squares). (Strains JJB31, JJB51, JJB87, JJB89, JJB99 and JJB101, respectively.) For clarity, only data from a subset of these strains are presented in each graph (as labeled) and, also for clarity, the *WT* and *15 nt* data is presented in all graphs. Note that (B) and (D) provide zoomed views of the data from the boxed areas of (A) and (C), respectively.
(TIF)

S3 Fig Misexpression of *sigG* from *^quad^*P*_sigG_* does not affect heat-resistant spore formation. The total number of heat-resistant spores per mL was determined for a wild type strain harboring unaltered *sigG* at its native locus (WT; strain PY79), a strain deleted for *sigG* (*ΔsigG*; strain AHB98), or strains deleted for *sigG* and harboring at the non-essential *ylnF* locus a copy of the *sigG* gene either under the control of wild type regulatory sequences (+*ylnF*::^WT^P_*sigG*_*-sigG*; strain LMB39) or regulatory sequences modified to remove or repair the four features identified in this study to dampen *sigG* expression (+*ylnF*::^*quad*^P_*sigG*_*-sigG*; strain AHB2819). Error bars indicate standard deviation based on three or more independent experiments.
(TIF)

S4 Fig Cells misexpressing *sigG* from *^quad^*P*_sigG_* and lacking *csfB* display defects in exponential and stationary phase vegetative growth. **(A)** Representative growth curves in liquid LB for cells expressing *sigG* from its wild type regulatory sequences (^WT^P_*sigG*_) or from regulatory sequences modified to remove or repair the four features identified in this study to dampen *sigG* expression (^*quad*^P_*sigG*_). Additionally, cells either harbored wild type *csfB*, or were deleted for the gene (*ΔcsfB*). (Strains EBM192 [^WT^P_*sigG*_; purple diamonds], EBM276 [^*quad*^P_*sigG*_; orange triangles], EBM282 [^WT^P_*sigG*_ *ΔcsfB*; blue squares], and EBM287 [^*quad*^P_*sigG*_-*sigG ΔcsfB*; green circles].) **(B)** Doubling time during exponential growth and maximal OD_600_ during stationary phase for each strain are reported as the average ± standard deviation based on three independent experiments. **p < 0.01, ****p < 0.0001, ns not significant, Student’s *t*-test.
(TIF)

S5 Fig Shortened *sigG* promoter element spacing and non-ATG start codon usage appears to be conserved in endospore-forming *Bacilli* but not *Clostridia*. Upstream regulatory sequences for *sigG* were retrieved from the genome sequences of representative endospore forming bacteria from the classes *Bacilli* and *Clostridia*. The sequence for the model organism *Bacillus subtilis* is boxed. The -35 and -10 promoter elements are bolded and shaded with gray boxes, and the consensus sequences for σ^F^ and σ^G^ are shown above [38]. The known transcription start site for the *B*. *subtilis sigG* transcript is also bolded. Known or putative ribosome binding sites are underlined. Shortened spacing between the -35 and -10 promoter elements as well as non-ATG start codons are indicated in red.
(TIF)

S6 Fig The ability of the *sigG* mRNA 5’ leader sequence to form a hairpin structure occluding the RBS appears to be conserved among endospore-forming *Bacilli*. Upstream regulatory sequences for *sigG* were retrieved from the genome sequences of representative endospore forming bacteria from the classes *Bacilli* and *Clostridia*. The 5’ mRNA leader sequences for each were queried for secondary structure by the RNAstructure method [43]. Potential secondary structures for a subset of the 5’ leader sequences are displayed using RNAbow diagrams [69], with base pairs are indicated by arcs, the thickness and shading of which is proportional to their probability. The partition function free energy (G) is indicated. The +1 transcription start site, RBS, and start codon for each are underlined. “+1*” indicates start sites selected based on alignment with the known *B*. *subtilis sigG* transcriptional start site.
(TIF)

S1 Table *B. subtilis* strains used in this study. (PDF)

S2 Table Plasmids used in this study. (PDF)

S3 Table Oligonucleotides used in this study. (PDF)

S4 Table Synthetic DNA fragments used in this study. (PDF)

S1 Text Supplemental materials and methods. (PDF)

## References

[1] Tan M. Temporal gene regulation during the *Chlamydial* developmental cycle. In: Tan M, Bavoil PM, editors. Intracellular Pathogens I: Chlamydiales. Washington, D.C; 2012 pp. 149–169.

[2] Hilbert DW, Piggot PJ. Compartmentalization of gene expression during *Bacillus subtilis* spore formation. Microbiol Mol Biol Rev. 2004;68: 234–262. doi: 10.1128/MMBR.68.2.234-262.2004 15187183 10.1128/MMBR.68.2.234-262.2004 PMC419919

[3] de Hoon MJL, Eichenberger P, Vitkup D. Hierarchical evolution of the bacterial sporulation network. Curr Biol. 2010;20: R735–R745. doi: 10.1016/j.cub.2010.06.031 20833318 10.1016/j.cub.2010.06.031 PMC2944226

[4] Arrieta-Ortiz ML, Hafemeister C, Bate AR, Chu T, Greenfield A, Shuster B, et al An experimentally supported model of the *Bacillus subtilis* global transcriptional regulatory network. Mol Syst Biol. 2015;11: 839–839. doi: 10.15252/msb.20156236 26577401 10.15252/msb.20156236 PMC4670728

[5] Li Z, Piggot PJ. Development of a two-part transcription probe to determine the completeness of temporal and spatial compartmentalization of gene expression during bacterial development. Proc Natl Acad Sci USA. 2001;98: 12538–12543. doi: 10.1073/pnas.221454798 11606741 10.1073/pnas.221454798 PMC60089

[6] Barák I, Wilkinson AJ. Where asymmetry in gene expression originates. Mol Microbiol. 2005;57: 611–620. doi: 10.1111/j.1365-2958.2005.04687.x 16045607 10.1111/j.1365-2958.2005.04687.x

[7] Schmidt R, Margolis P, Duncan L, Coppolecchia R, Moran CP, Losick R. Control of developmental transcription factor σ^F^ by sporulation regulatory proteins SpoIIAA and SpoIIAB in *Bacillus subtilis*. Proc Natl Acad Sci USA. 1990;87: 9221–9225. 2123551 10.1073/pnas.87.23.9221 PMC55136

[8] Partridge SR, Foulger D, Errington J. The role of σ^F^ in prespore-specific transcription in *Bacillus subtilis*. Mol Microbiol. 1991;5: 757–767. 1904527 10.1111/j.1365-2958.1991.tb00746.x

[9] Sun DX, Cabrera-Martinez RM, Setlow P. Control of transcription of the *Bacillus subtilis spoIIIG* gene, which codes for the forespore-specific transcription factor σ^G^. J Bacteriol. 1991;173: 2977–2984. 1902213 10.1128/jb.173.9.2977-2984.1991 PMC207881

[10] Camp AH, Wang AF, Losick R. A small protein required for the switch from σ^F^ to σ^G^ during sporulation in *Bacillus subtilis*. J Bacteriol. 2011;193: 116–124. doi: 10.1128/JB.00949-10 21037003 10.1128/JB.00949-10 PMC3019954

[11] Wang Erickson AF, Deighan P, Chen S, Barrasso K, Garcia CP, Martínez-Lumbreras S, et al A novel RNA polymerase-binding protein that interacts with a sigma-factor docking site. Mol Microbiol. 2017;105: 652–662. doi: 10.1111/mmi.13724 28598017 10.1111/mmi.13724 PMC5558796

[12] Crawshaw AD, Serrano M, Stanley WA, Henriques AO, Salgado PS. A mother cell-to-forespore channel: current understanding and future challenges. FEMS Microbiol Lett. 2014;358: 129–136. doi: 10.1111/1574-6968.12554 25105965 10.1111/1574-6968.12554

[13] Camp AH, Losick R. A feeding tube model for activation of a cell-specific transcription factor during sporulation in *Bacillus subtilis*. Genes Dev. 2009;23: 1014–1024. doi: 10.1101/gad.1781709 19390092 10.1101/gad.1781709 PMC2675869

[14] Doan T, Morlot C, Meisner J, Serrano M, Henriques AO, Moran CP, et al Novel secretion apparatus maintains spore integrity and developmental gene expression in *Bacillus subtilis*. PLoS Genet. 2009;5: e1000566 doi: 10.1371/journal.pgen.1000566 19609349 10.1371/journal.pgen.1000566 PMC2703783

[15] Decatur A, Losick R. Identification of additional genes under the control of the transcription factor σ^F^ of *Bacillus subtilis*. J Bacteriol. 1996;178: 5039–5041. 8759874 10.1128/jb.178.16.5039-5041.1996 PMC178293

[16] Chary VK, Xenopoulos P, Piggot PJ. Expression of the σ^F^-directed *csfB* locus prevents premature appearance of σ^G^ activity during sporulation of *Bacillus subtilis*. J Bacteriol. 2007;189: 8754–8757. doi: 10.1128/JB.01265-07 17921305 10.1128/JB.01265-07 PMC2168958

[17] Karmazyn-Campelli C, Rhayat L, Carballido-López R, Duperrier S, Frandsen N, Stragier P. How the early sporulation sigma factor σ^F^ delays the switch to late development in *Bacillus subtilis*. Mol Microbiol. 2008;67: 1169–1180. doi: 10.1111/j.1365-2958.2008.06121.x 18208527 10.1111/j.1365-2958.2008.06121.x

[18] Rhayat L, Duperrier S, Carballido-López R, Pellegrini O, Stragier P. Genetic dissection of an inhibitor of the sporulation sigma factor σ^G^. J Mol Biol. 2009;390: 835–844. doi: 10.1016/j.jmb.2009.05.073 19497328 10.1016/j.jmb.2009.05.073

[19] Camp AH, Losick R. A novel pathway of intercellular signalling in *Bacillus subtilis* involves a protein with similarity to a component of type III secretion channels. Mol Microbiol. 2008;69: 402–417. doi: 10.1111/j.1365-2958.2008.06289.x 18485064 10.1111/j.1365-2958.2008.06289.x PMC2574792

[20] Partridge SR, Errington J. The importance of morphological events and intercellular interactions in the regulation of prespore-specific gene expression during sporulation in *Bacillus subtilis*. Mol Microbiol. 1993;8: 945–955. 8355618 10.1111/j.1365-2958.1993.tb01639.x

[21] Karow ML, Piggot PJ. Construction of *gusA* transcriptional fusion vectors for *Bacillus subtilis* and their utilization for studies of spore formation. Gene. 1995;163: 69–74. 7557481 10.1016/0378-1119(95)00402-r

[22] Sun YL, Sharp MD, Pogliano K. A dispensable role for forespore-specific gene expression in engulfment of the forespore during sporulation of *Bacillus subtilis*. J Bacteriol. 2000;182: 2919–2927. 10781563 10.1128/jb.182.10.2919-2927.2000 PMC102003

[23] Evans L, Feucht A, Errington J. Genetic analysis of the *Bacillus subtilis sigG* promoter, which controls the sporulation-specific transcription factor σ^G^. Microbiology. 2004;150: 2277–2287. doi: 10.1099/mic.0.26914-0 15256570 10.1099/mic.0.26914-0

[24] Stragier P, Losick R. Molecular genetics of sporulation in *Bacillus subtilis*. Annu Rev Genet. 1996;30: 297–341. doi: 10.1146/annurev.genet.30.1.297 8982457 10.1146/annurev.genet.30.1.297

[25] Karow ML, Glaser P, Piggot PJ. Identification of a gene, *spoIIR*, that links the activation of σ^E^ to the transcriptional activity of σ^F^ during sporulation in *Bacillus subtilis*. Proc Natl Acad Sci USA. 1995;92: 2012–2016. 7892217 10.1073/pnas.92.6.2012 PMC42413

[26] Londoño-Vallejo JA, Stragier P. Cell-cell signaling pathway activating a developmental transcription factor in *Bacillus subtilis*. Genes Dev. 1995;9: 503–508. 7883171 10.1101/gad.9.4.503

[27] Londoño-Vallejo JA, Fréhel C, Stragier P. SpoIIQ, a forespore-expressed gene required for engulfment in *Bacillus subtilis*. Mol Microbiol. 1997;24: 29–39. 9140963 10.1046/j.1365-2958.1997.3181680.x

[28] Serrano M, Neves A, Soares CM, Moran CP, Henriques AO. Role of the anti-sigma factor SpoIIAB in regulation of σ^G^ during *Bacillus subtilis* sporulation. J Bacteriol. 2004;186: 4000–4013. doi: 10.1128/JB.186.12.4000-4013.2004 15175314 10.1128/JB.186.12.4000-4013.2004 PMC419951

[29] Sun D, Fajardo-Cavazos P, Sussman MD, Tovar-Rojo F, Cabrera-Martinez RM, Setlow P. Effect of chromosome location of *Bacillus subtilis* forespore genes on their *spo* gene dependence and transcription by Eσ^F^: identification of features of good Eσ^F^-dependent promoters. J Bacteriol. 1991;173: 7867–7874. 1744043 10.1128/jb.173.24.7867-7874.1991 PMC212578

[30] Vellanoweth RL, Rabinowitz JC. The influence of ribosome-binding-site elements on translational efficiency in *Bacillus subtilis* and *Escherichia coli in vivo*. Mol Microbiol. 1992;6: 1105–1114. 1375309 10.1111/j.1365-2958.1992.tb01548.x

[31] Tuller T, Zur H. Multiple roles of the coding sequence 5' end in gene expression regulation. Nucleic Acids Res. 2015;43: 13–28. doi: 10.1093/nar/gku1313 25505165 10.1093/nar/gku1313 PMC4288200

[32] Gaspar P, Moura G, Santos MAS, Oliveira JL. mRNA secondary structure optimization using a correlated stem-loop prediction. Nucleic Acids Res. 2013;41: e73 doi: 10.1093/nar/gks1473 23325845 10.1093/nar/gks1473 PMC3616703

[33] Veening J-W, Smits WK, Hamoen LW, Jongbloed JDH, Kuipers OP. Visualization of differential gene expression by improved cyan fluorescent protein and yellow fluorescent protein production in *Bacillus subtilis*. Appl Environ Microbiol. 2004;70: 6809–6815. doi: 10.1128/AEM.70.11.6809-6815.2004 15528548 10.1128/AEM.70.11.6809-6815.2004 PMC525234

[34] Foulger D, Errington J. The role of the sporulation gene *spoIIIE* in the regulation of prespore-specific gene expression in *Bacillus subtilis*. Mol Microbiol. 1989;3: 1247–1255. 2507870 10.1111/j.1365-2958.1989.tb00275.x

[35] Smith K, Bayer ME, Youngman P. Physical and functional characterization of the *Bacillus subtilis spoIIM* gene. J Bacteriol. 1993;175: 3607–3617. 8501064 10.1128/jb.175.11.3607-3617.1993 PMC204762

[36] Karmazyn-Campelli C, Bonamy C, Savelli B, Stragier P. Tandem genes encoding σ-factors for consecutive steps of development in *Bacillus subtilis*. Genes Dev. 1989;3: 150–157. 2497052 10.1101/gad.3.2.150

[37] Steil L, Serrano M, Henriques AO, Völker U. Genome-wide analysis of temporally regulated and compartment-specific gene expression in sporulating cells of *Bacillus subtilis*. Microbiology. 2005;151: 399–420. doi: 10.1099/mic.0.27493-0 15699190 10.1099/mic.0.27493-0

[38] Wang ST, Setlow B, Conlon EM, Lyon JL, Imamura D, Sato T, et al The forespore line of gene expression in *Bacillus subtilis*. J Mol Biol. 2006;358: 16–37. doi: 10.1016/j.jmb.2006.01.059 16497325 10.1016/j.jmb.2006.01.059

[39] Bagyan I, Casillas-Martinez L, Setlow P. The *katX* gene, which codes for the catalase in spores of *Bacillus subtilis*, is a forespore-specific gene controlled by σ^F^, and KatX is essential for hydrogen peroxide resistance of the germinating spore. J Bacteriol. 1998;180: 2057–2062. 9555886 10.1128/jb.180.8.2057-2062.1998 PMC107130

[40] Lewis PJ, Partridge SR, Errington J. σ factors, asymmetry, and the determination of cell fate in *Bacillus subtilis*. Proc Natl Acad Sci USA. 1994;91: 3849–3853. 8171000 10.1073/pnas.91.9.3849 PMC43679

[41] Sierro N, Makita Y, de Hoon M, Nakai K. DBTBS: a database of transcriptional regulation in *Bacillus subtilis* containing upstream intergenic conservation information. Nucleic Acids Res. 2008;36: D93–D96. doi: 10.1093/nar/gkm910 17962296 10.1093/nar/gkm910 PMC2247474

[42] Meyer MM. The role of mRNA structure in bacterial translational regulation. Wiley Interdiscip Rev RNA. 2017;8: e1370 doi: 10.1002/wrna.1370 27301829 10.1002/wrna.1370

[43] Reuter JS, Mathews DH. RNAstructure: software for RNA secondary structure prediction and analysis. BMC Bioinformatics. 2010;11: 129 doi: 10.1186/1471-2105-11-129 20230624 10.1186/1471-2105-11-129 PMC2984261

[44] Serrano M, Real G, Santos J, Carneiro J, Moran CP, Henriques AO. A negative feedback loop that limits the ectopic activation of a cell type-specific sporulation sigma factor of *Bacillus subtilis*. PLoS Genet. 2011;7: e1002220 doi: 10.1371/journal.pgen.1002220 21935351 10.1371/journal.pgen.1002220 PMC3174212

[45] Stefano JE, Gralla JD. Spacer mutations in the *lac* p^s^ promoter. Proc Natl Acad Sci USA. 1982;79: 1069–1072. 6951162 10.1073/pnas.79.4.1069 PMC345901

[46] Russell DR, Bennett GN. Construction and analysis of in vivo activity of *E*. *coli* promoter hybrids and promoter mutants that alter the -35 to -10 spacing. Gene. 1982;20: 231–243. 6299890 10.1016/0378-1119(82)90042-7

[47] Aoyama T, Takanami M, Ohtsuka E, Taniyama Y, Marumoto R, Sato H, et al Essential structure of *E*. *coli* promoter: effect of spacer length between the two consensus sequences on promoter function. Nucleic Acids Res. 1983;11: 5855–5864. 6310517 10.1093/nar/11.17.5855 PMC326322

[48] Mulligan ME, Brosius J, McClure WR. Characterization *in vitro* of the effect of spacer length on the activity of *Escherichia coli* RNA polymerase at the TAC promoter. J Biol Chem. 1985;260: 3529–3538. 3882710

[49] Kudla G, Murray AW, Tollervey D, Plotkin JB. Coding-sequence determinants of gene expression in *Escherichia coli*. Science. 2009;324: 255–258. doi: 10.1126/science.1170160 19359587 10.1126/science.1170160 PMC3902468

[50] Goodman DB, Church GM, Kosuri S. Causes and effects of N-terminal codon bias in bacterial genes. Science. 2013;342: 475–479. doi: 10.1126/science.1241934 24072823 10.1126/science.1241934

[51] Allert M, Cox JC, Hellinga HW. Multifactorial determinants of protein expression in prokaryotic open reading frames. J Mol Biol. 2010;402: 905–918. doi: 10.1016/j.jmb.2010.08.010 20727358 10.1016/j.jmb.2010.08.010 PMC2963087

[52] Masuda ES, Anaguchi H, Yamada K, Kobayashi Y. Two developmental genes encoding σ factor homologs are arranged in tandem in *Bacillus subtilis*. Proc Natl Acad Sci USA. 1988;85: 7637–7641. 2459711 10.1073/pnas.85.20.7637 PMC282247

[53] Mukherjee S, Sengupta S. Riboswitch Scanner: an efficient pHMM-based web-server to detect riboswitches in genomic sequences. Bioinformatics. 2016;32: 776–778. doi: 10.1093/bioinformatics/btv640 26519506 10.1093/bioinformatics/btv640

[54] Abreu-Goodger C, Merino E. RibEx: a web server for locating riboswitches and other conserved bacterial regulatory elements. Nucleic Acids Res. 2005;33: W690–W692. doi: 10.1093/nar/gki445 15980564 10.1093/nar/gki445 PMC1160206

[55] Chang T-H, Huang H-D, Wu L-C, Yeh C-T, Liu B-J, Horng J-T. Computational identification of riboswitches based on RNA conserved functional sequences and conformations. RNA. 2009;15: 1426–1430. doi: 10.1261/rna.1623809 19460868 10.1261/rna.1623809 PMC2704089

[56] Schmidt R, Decatur AL, Rather PN, Moran CP, Losick R. *Bacillus subtilis* Lon protease prevents inappropriate transcription of genes under the control of the sporulation transcription factor σ^G^. J Bacteriol. 1994;176: 6528–6537. 7961403 10.1128/jb.176.21.6528-6537.1994 PMC197006

[57] Serrano M, Hövel S, Moran CP, Henriques AO, Völker U. Forespore-specific transcription of the *lonB* gene during sporulation in *Bacillus subtilis*. J Bacteriol. 2001;183: 2995–3003. doi: 10.1128/JB.183.10.2995-3003.2001 11325926 10.1128/JB.183.10.2995-3003.2001 PMC95198

[58] Rather PN, Coppolecchia R, DeGrazia H, Moran CP. Negative regulator of σ^G^-controlled gene expression in stationary-phase *Bacillus subtilis*. J Bacteriol. 1990;172: 709–715. 2105300 10.1128/jb.172.2.709-715.1990 PMC208497

[59] Serrano M, Gao J, Bota J, Bate AR, Meisner J, Eichenberger P, et al Dual-specificity anti-sigma factor reinforces control of cell-type specific gene expression in *Bacillus subtilis*. PLoS Genet. 2015;11: e1005104 doi: 10.1371/journal.pgen.1005104 25835496 10.1371/journal.pgen.1005104 PMC4383634

[60] Flanagan KA, Comber JD, Mearls E, Fenton C, Wang Erickson AF, Camp AH. A membrane-embedded amino acid couples the SpoIIQ channel protein to anti-sigma factor transcriptional repression during *Bacillus subtilis* sporulation. J Bacteriol. 2016;198: 1451–1463. doi: 10.1128/JB.00958-15 26929302 10.1128/JB.00958-15 PMC4836239

[61] Kirchman PA, DeGrazia H, Kellner EM, Moran CP. Forespore-specific disappearance of the sigma-factor antagonist SpoIIAB: implications for its role in determination of cell fate in *Bacillus subtilis*. Mol Microbiol. 1993;8: 663–671. 8332059 10.1111/j.1365-2958.1993.tb01610.x

[62] Youngman P, Perkins JB, Losick R. Construction of a cloning site near one end of Tn917 into which foreign DNA may be inserted without affecting transposition in *Bacillus subtilis* or expression of the transposon-borne *erm* gene. Plasmid. 1984;12: 1–9. 6093169 10.1016/0147-619x(84)90061-1

[63] Schaeffer P, Millet J, Aubert JP. Catabolic repression of bacterial sporulation. Proc Natl Acad Sci USA. 1965;54: 704–711. 4956288 10.1073/pnas.54.3.704 PMC219731

[64] Nicholson WL, Setlow P. Sporulation, germination and outgrowth. In: Harwood CR, Cutting SM, editors. Molecular Biology Methods for *Bacillus*. New York City, NY; 1990 pp. 391–450.

[65] Sterlini JM, Mandelstam J. Commitment to sporulation in *Bacillus subtilis* and its relationship to development of actinomycin resistance. Biochem J. 1969;113: 29–37. 4185146 10.1042/bj1130029 PMC1184601

[66] Schneider CA, Rasband WS, Eliceiri KW. NIH Image to ImageJ: 25 years of image analysis. Nat Methods. 2012;9: 671–675. 22930834 10.1038/nmeth.2089 PMC5554542

[67] Schindelin J, Arganda-Carreras I, Frise E, Kaynig V, Longair M, Pietzsch T, et al Fiji: an open-source platform for biological-image analysis. Nat Methods. 2012;9: 676–682. doi: 10.1038/nmeth.2019 22743772 10.1038/nmeth.2019 PMC3855844

[68] Gruber AR, Bernhart SH, Lorenz R. The ViennaRNA web services. Methods Mol Biol. 2015;1269: 307–326. doi: 10.1007/978-1-4939-2291-8_19 25577387 10.1007/978-1-4939-2291-8_19

[69] Aalberts DP, Jannen WK. Visualizing RNA base-pairing probabilities with RNAbow diagrams. RNA. 2013;19: 475–478. doi: 10.1261/rna.033365.112 23407410 10.1261/rna.033365.112 PMC3677257

