# Supplementary figures and images for: Transcription and translation of the sigG gene is tuned for proper execution of the switch from early to late gene expression in the developing Bacillus subtilis spore
Source: PLoS Genet. 2018 Apr 27;14(4):e1007350. doi: 10.1371/journal.pgen.1007350 (PMC5942855; doi:10.1371/journal.pgen.1007350)

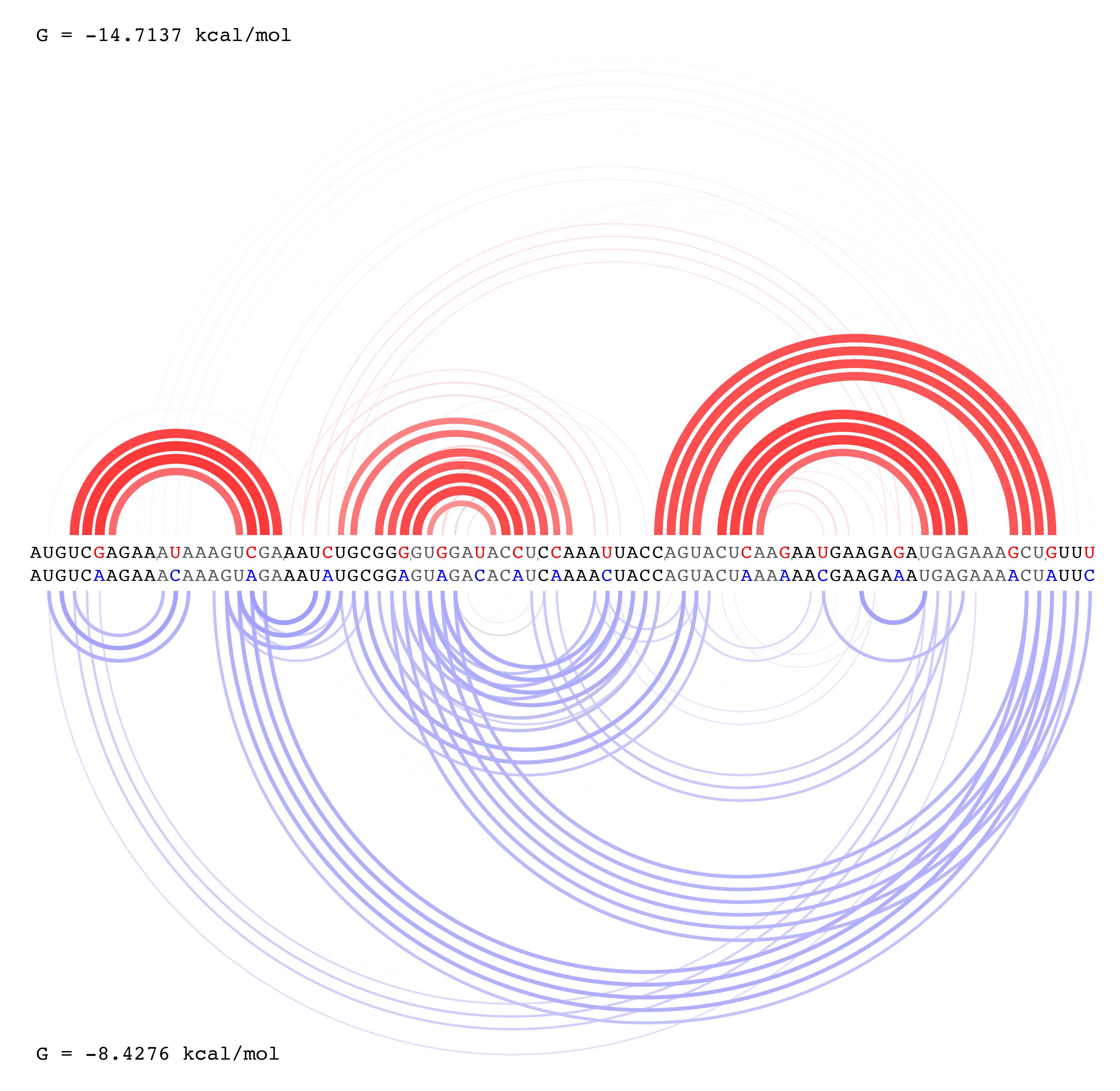

Supplement: S1 Fig — An AUG (ATG) start codon followed by codons 2–28 of B. subtilis sigG are shown (top sequence), as is a sequence comprised of synonymous codons selected by the mRNA Optimizer tool [32] to minimize secondary structure (bottom sequence). Note that the latter is the sequence present in the PsigG-ATG-RSSsigG2-28-lacZ reporter construct used in Fig 2. Nucleotide differences are indicated in red and blue text. Both sequences were analyzed for secondary structure by the RNAstructure method [43]. Potential secondary structures are displayed above (red) or below (blue) using RNAbow diagrams [69], with base pairs indicated by arcs, the thickness and shading of which is proportional to their probability. The partition function free energy (G) for each is indicated. (TIF) [file pgen.1007350.s001.tif]

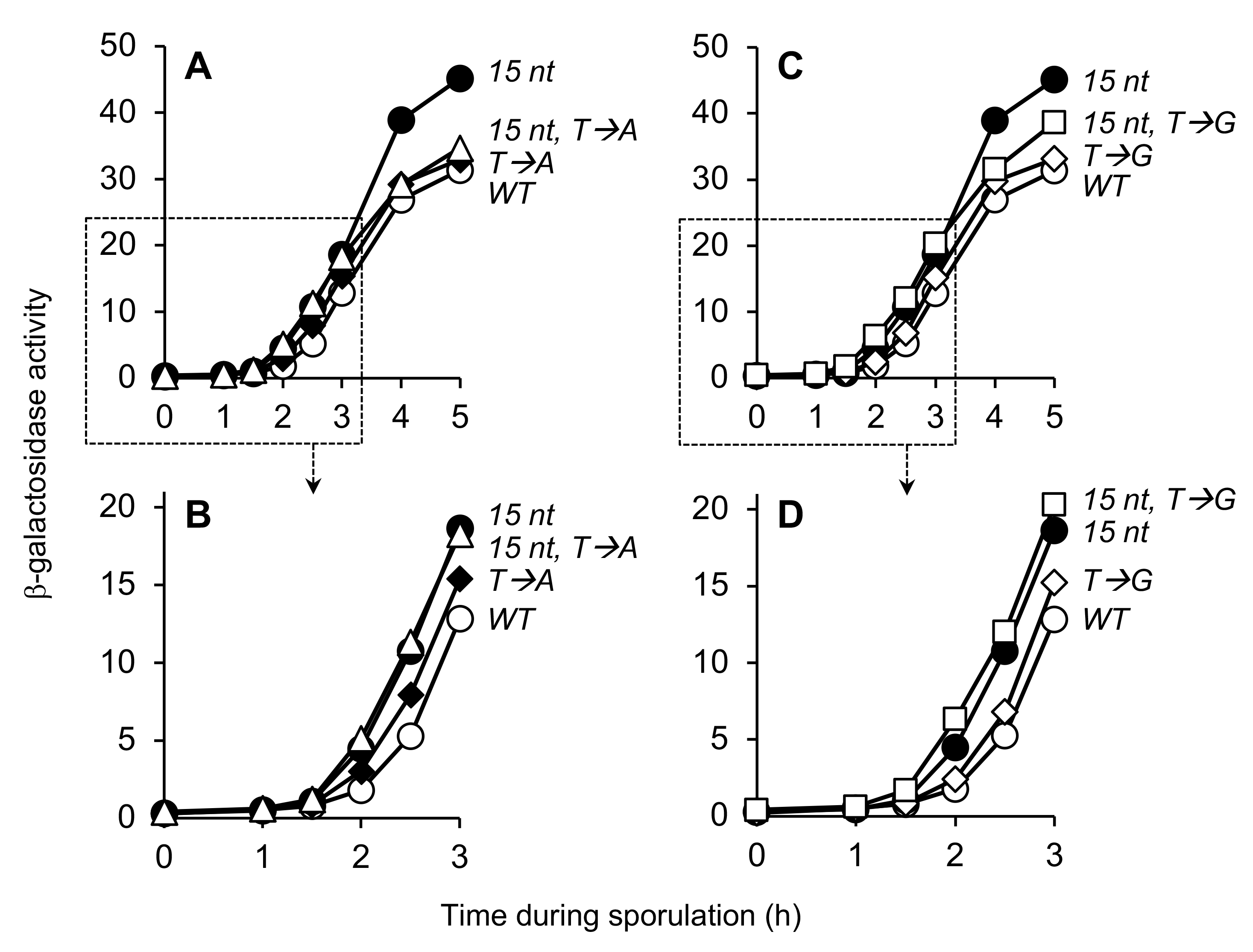

Supplement: S2 Fig — β-Galactosidase production was monitored during sporulation of strains harboring PsigG-lacZ (WT; open circles), 15ntPsigG-lacZ (15 nt; closed circles), T→APsigG-lacZ (T→A; open diamonds), T→GPsigG-lacZ (T→G; closed diamonds) 15nt,T→APsigG-lacZ (15 nt, T→A; open triangles), and 15nt,T→GPsigG-lacZ (15 nt, T→G; open squares). (Strains JJB31, JJB51, JJB87, JJB89, JJB99 and JJB101, respectively.) For clarity, only data from a subset of these strains are presented in each graph (as labeled) and, also for clarity, the WT and 15 nt data is presented in all graphs. Note that (B) and (D) provide zoomed views of the data from the boxed areas of (A) and (C), respectively. (TIF) [file pgen.1007350.s002.tif]

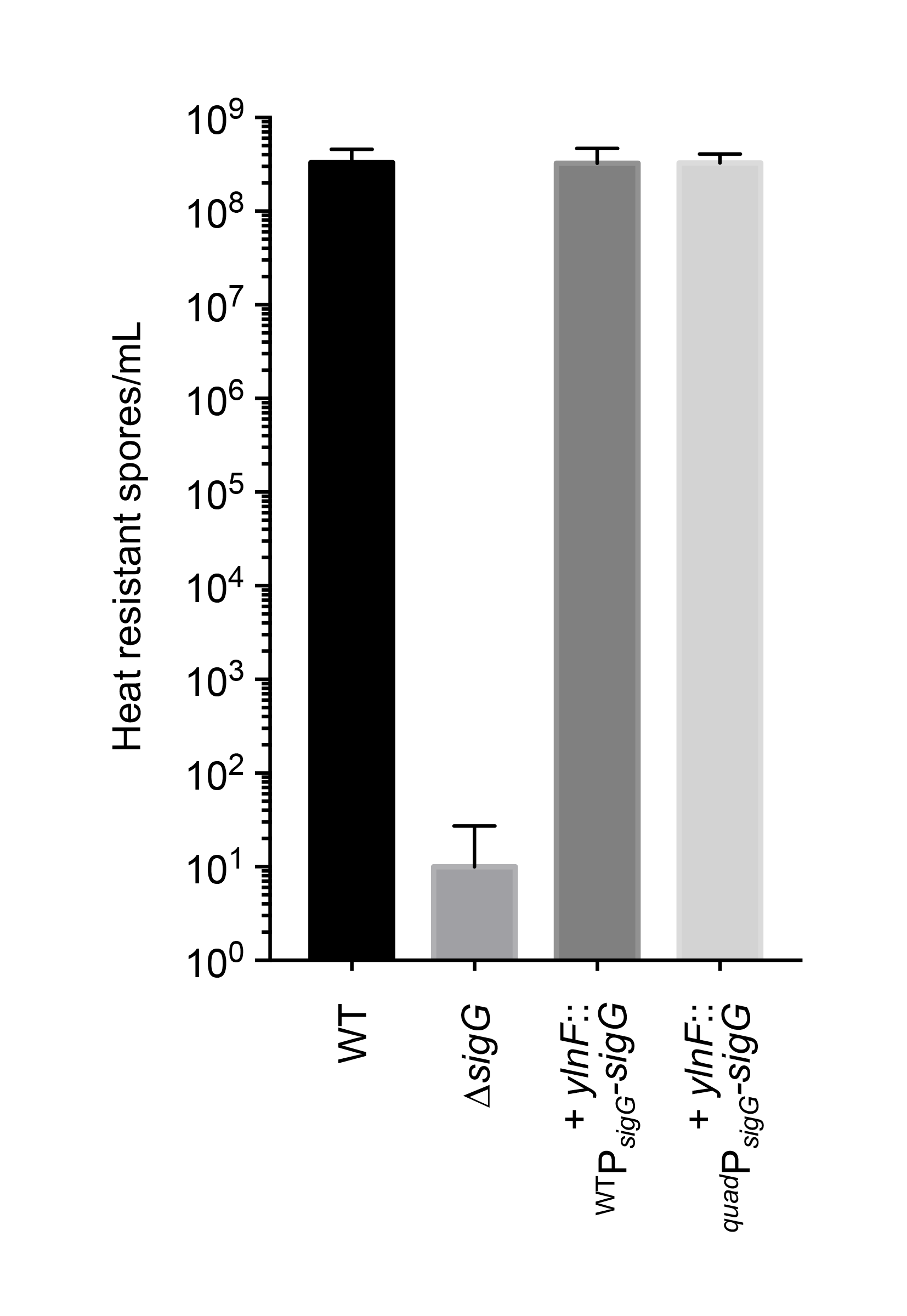

Supplement: S3 Fig — The total number of heat-resistant spores per mL was determined for a wild type strain harboring unaltered sigG at its native locus (WT; strain PY79), a strain deleted for sigG (ΔsigG; strain AHB98), or strains deleted for sigG and harboring at the non-essential ylnF locus a copy of the sigG gene either under the control of wild type regulatory sequences (+ylnF::WTPsigG-sigG; strain LMB39) or regulatory sequences modified to remove or repair the four features identified in this study to dampen sigG expression (+ylnF::quadPsigG-sigG; strain AHB2819). Error bars indicate standard deviation based on three or more independent experiments. (TIF) [file pgen.1007350.s003.tif]

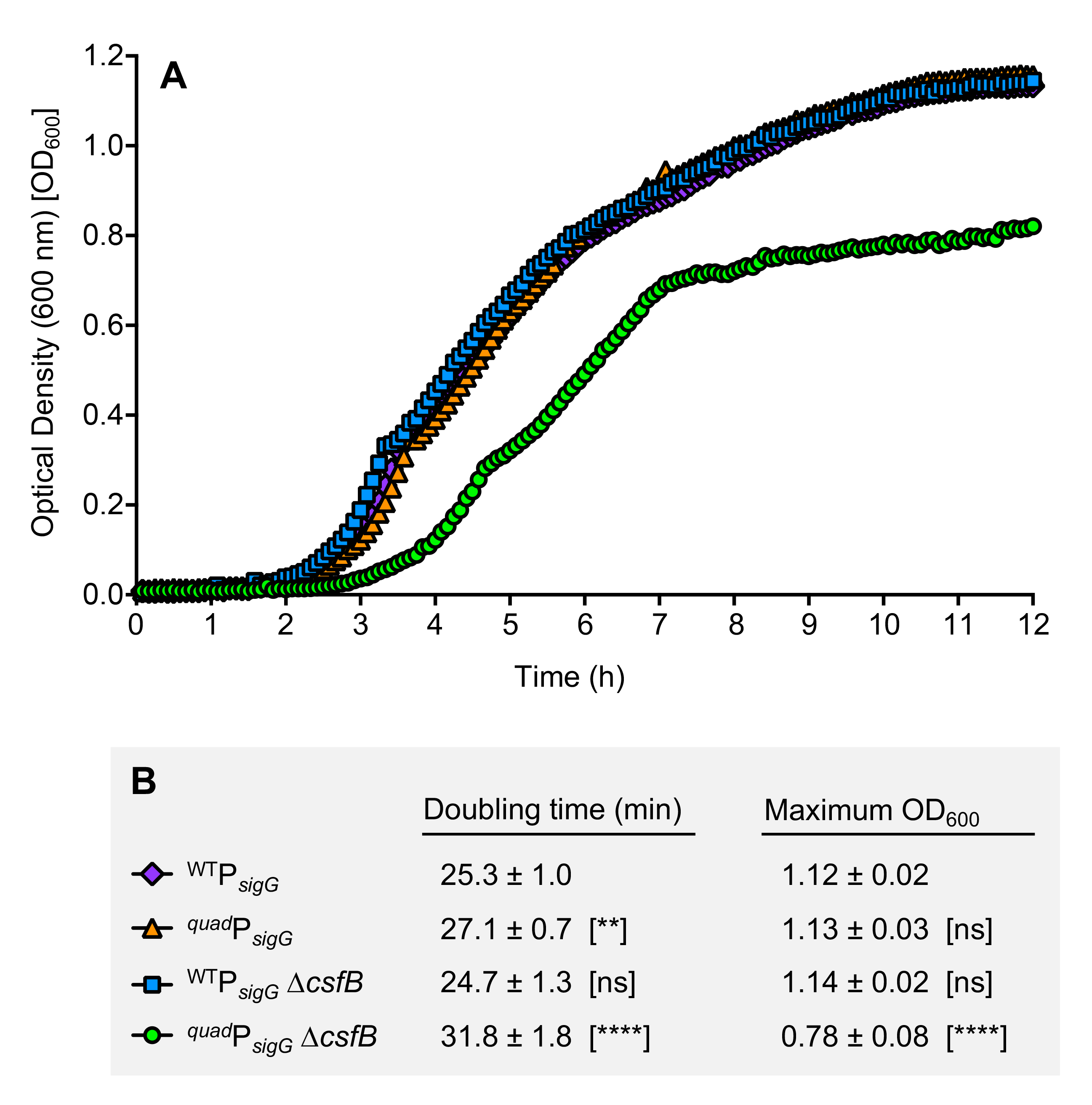

Supplement: S4 Fig — (A) Representative growth curves in liquid LB for cells expressing sigG from its wild type regulatory sequences (WTPsigG) or from regulatory sequences modified to remove or repair the four features identified in this study to dampen sigG expression (quadPsigG). Additionally, cells either harbored wild type csfB, or were deleted for the gene (ΔcsfB). (Strains EBM192 [WTPsigG; purple diamonds], EBM276 [quadPsigG; orange triangles], EBM282 [WTPsigG ΔcsfB; blue squares], and EBM287 [quadPsigG-sigG ΔcsfB; green circles].) (B) Doubling time during exponential growth and maximal OD600 during stationary phase for each strain are reported as the average ± standard deviation based on three independent experiments. **p < 0.01, ****p < 0.0001, ns not significant, Student’s t-test. (TIF) [file pgen.1007350.s004.tif]

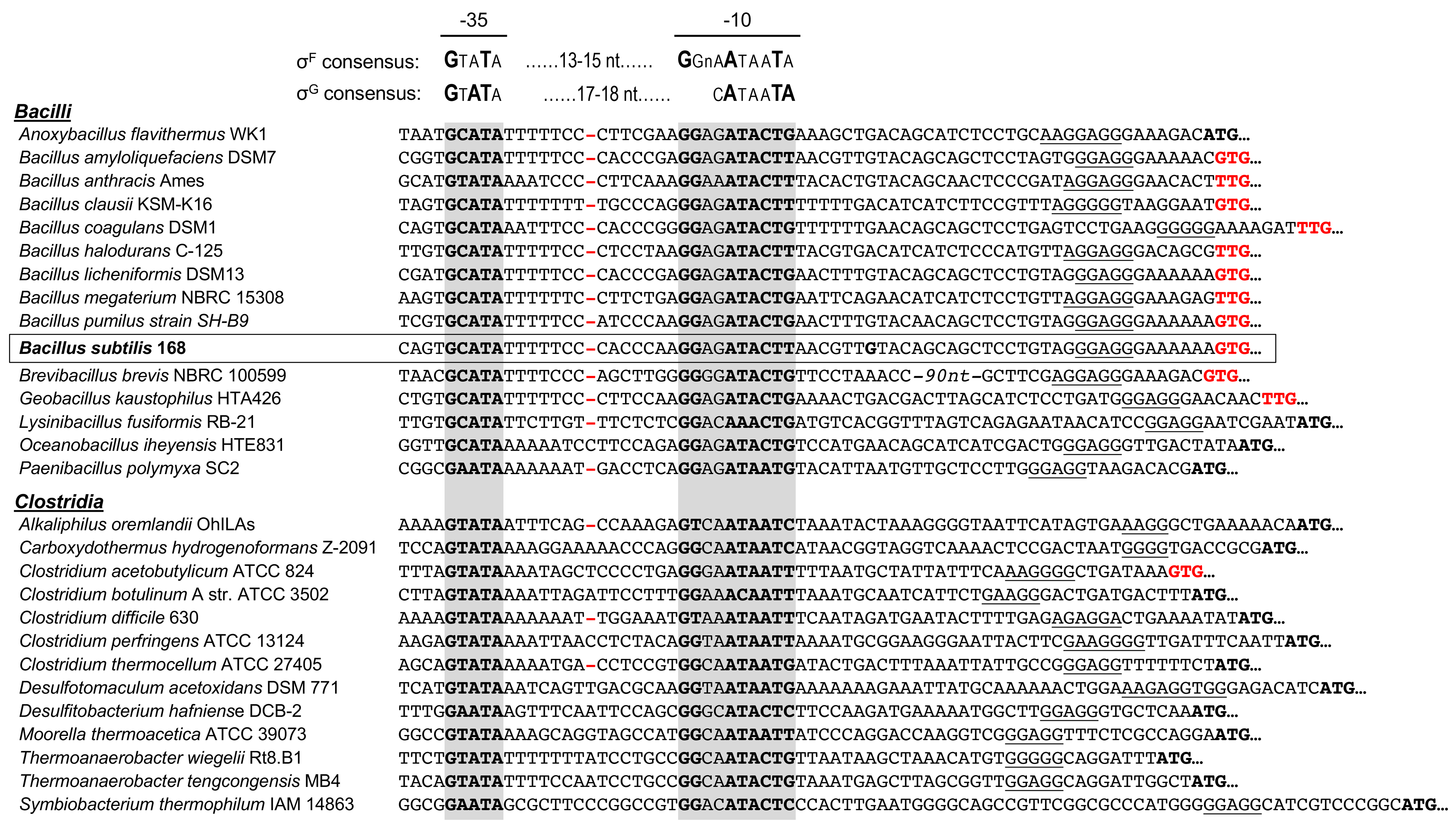

Supplement: S5 Fig — Upstream regulatory sequences for sigG were retrieved from the genome sequences of representative endospore forming bacteria from the classes Bacilli and Clostridia. The sequence for the model organism Bacillus subtilis is boxed. The -35 and -10 promoter elements are bolded and shaded with gray boxes, and the consensus sequences for σF and σG are shown above [38]. The known transcription start site for the B. subtilis sigG transcript is also bolded. Known or putative ribosome binding sites are underlined. Shortened spacing between the -35 and -10 promoter elements as well as non-ATG start codons are indicated in red. (TIF) [file pgen.1007350.s005.tif]

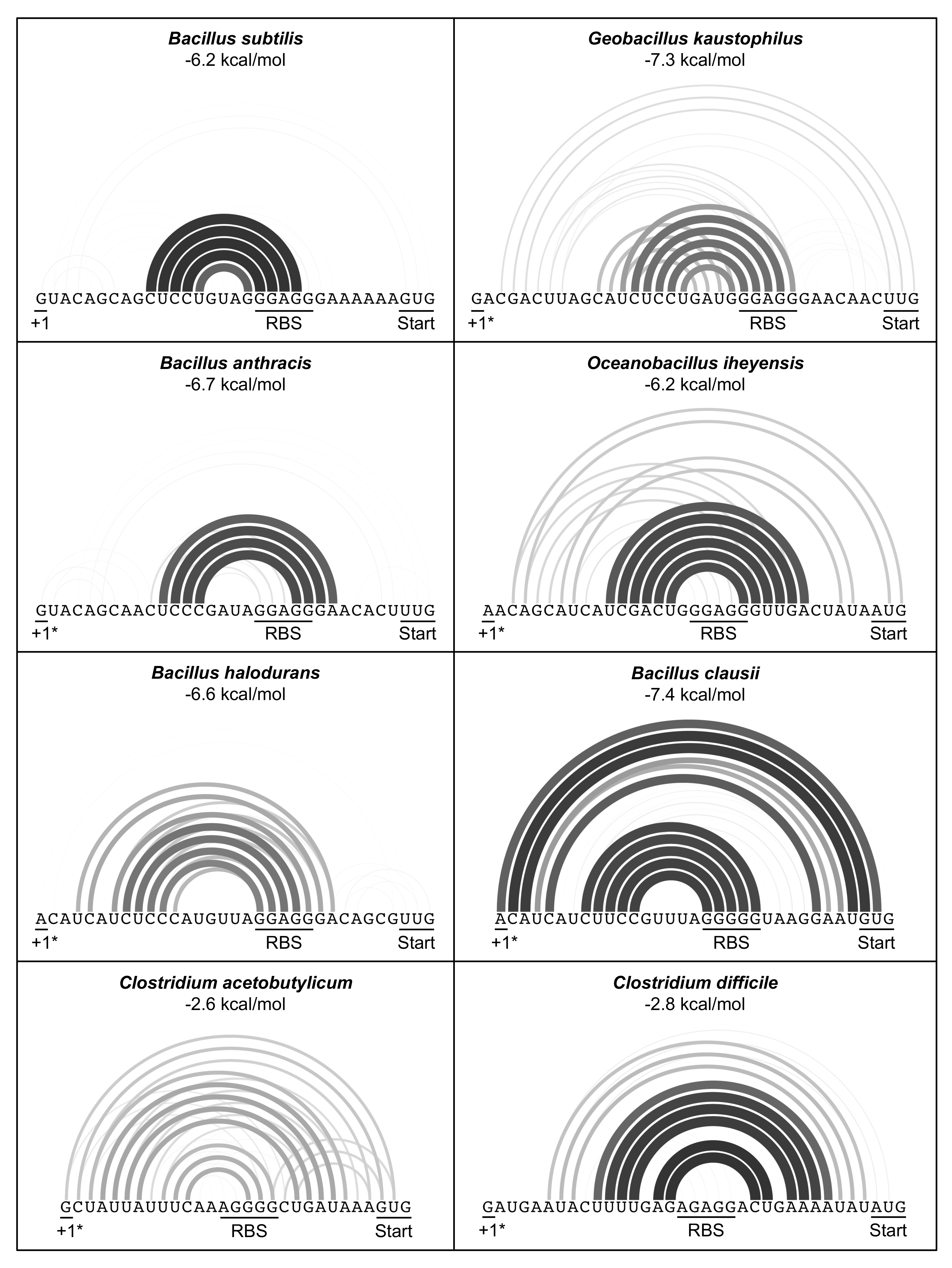

Supplement: S6 Fig — Upstream regulatory sequences for sigG were retrieved from the genome sequences of representative endospore forming bacteria from the classes Bacilli and Clostridia. The 5’ mRNA leader sequences for each were queried for secondary structure by the RNAstructure method [43]. Potential secondary structures for a subset of the 5’ leader sequences are displayed using RNAbow diagrams [69], with base pairs are indicated by arcs, the thickness and shading of which is proportional to their probability. The partition function free energy (G) is indicated. The +1 transcription start site, RBS, and start codon for each are underlined. “+1*” indicates start sites selected based on alignment with the known B. subtilis sigG transcriptional start site. (TIF) [file pgen.1007350.s006.tif]
