# Supplementary material for: Transcription and translation of the sigG gene is tuned for proper execution of the switch from early to late gene expression in the developing Bacillus subtilis spore
Source: PLoS Genet. 2018 Apr 27;14(4):e1007350. doi: 10.1371/journal.pgen.1007350 (PMC5942855; doi:10.1371/journal.pgen.1007350)
Supplement: S1 Table — (PDF) [file pgen.1007350.s007.pdf]

S1 Table. *B. subtilis* strains used in this study

Strain	Genotype[a,b]	Source or reference
PY79	Prototrophic wild type	[62]
AHB98	Δ*sigG*::*kan*	[19]
AHB317	*amyE*::P_{sspB}-*lacZ cat*	This study
AHB881	*amyE*::P_{spoIIQ}-*lacZ cat*	[13]
AHB882	*amyE*::P_{spoIIQ}-*lacZ cat* Δ*sigG*::*kan*	[13]
AHB883	*amyE*::$P_{sigG}^{+10\rightarrow+30}$-*ATG-lacZ cat*	This study
AHB915	*amyE*::P_{spoIIQ}-*lacZ cat* Δ*sigG*::*kan* Δ*sigF*::*erm*	[13]
AHB916	*amyE*::P_{spoIIQ}-*lacZ cat* Δ*sigG*::*kan* Δ*spoIIQ*::*erm*	[13]
AHB917	*amyE*::P_{spoIIQ}-*lacZ cat* Δ*sigE-sigG*::*erm*	This study
AHB1017	*amyE*::P_{spoIIQ}-*lacZ cat* Δ*sigG*::*kan* Δ*spoIIIAA-AH*::*erm*	[13]
AHB1274	*amyE*::P_{sigG}-*ATG-lacZ cat*	This study
AHB2124	*amyE*::$P_{sigG}^{+24\rightarrow+30}$-*ATG-lacZ cat*	This study
AHB2126	*amyE*::$P_{sigG}^{+10\rightarrow+18}$-*ATG-lacZ cat*	This study
AHB2819	Δ*sigG*::*kan ylnF*::Tn*917*::*amyE*::$^{15nt,mut7}P_{sigG}$-*ATG*-^{RSS}*sigG* ("$^{quad}P_{sigG}$-*sigG*") *spc*	This study
AM3	*amyE*::P_{sigG}-*spacer-RBS-ATG-lacZ cat*	This study
AM4	*amyE*::$P_{sigG}^{+10\rightarrow+15}$-*spacer-RBS-ATG-lacZ cat*	This study
CFB429	Δ*sigG*::*kan ylnF*::Tn*917*::*amyE*::P_{sigG}-*sigG spc amyE*::P_{spoIIQ}-*lacZ cat*	This study
CFB431	Δ*sigG*::*kan ylnF*::Tn*917*::*amyE*::$^{15nt,mut7}P_{sigG}$-*ATG*-^{RSS}*sigG* ("$^{quad}P_{sigG}$-*sigG*") *spc amyE*::P_{spoIIQ}-*lacZ cat*	This study
CFB435	Δ*sigG*::*kan ylnF*::Tn*917*::*amyE*::P_{sigG}-*sigG spc amyE*::P_{sspB}-*lacZ cat*	This study
CFB437	Δ*sigG*::*kan ylnF*::Tn*917*::*amyE*::$^{15nt,mut7}P_{sigG}$-*ATG*-^{RSS}*sigG* ("$^{quad}P_{sigG}$-*sigG*") *spc amyE*::P_{sspB}-*lacZ cat*	This study
EBM175	*amyE*::P_{sigG}-*ATG-sigG*$^{2\text{-}28}$-*lacZ cat*	This study
EBM177	*amyE*::P_{sigG}-*sigG*$^{1\text{-}28}$-*lacZ cat*	This study
EBM192	Δ*sigG*::*kan ylnF*::Tn*917*::*amyE*::P_{sigG}-*sigG spc amyE*::P_{sspB}-*gfp cat*	This study
EBM237	*amyE*::P_{sigG}-*ATG*-^{RSS}*sigG*$^{2\text{-}28}$-*lacZ cat*	This study
EBM262	*amyE*::$^{15nt}P_{sigG}^{mut7}$-*ATG*-^{RSS}*sigG*$^{2\text{-}28}$-*lacZ* ("$^{quad}P_{sigG}$-*lacZ*") *cat*	This study
EBM276	Δ*sigG*::*kan ylnF*::Tn*917*::*amyE*::$^{15nt,mut7}P_{sigG}$-*ATG*-^{RSS}*sigG* ("$^{quad}P_{sigG}$-*sigG*") *spc amyE*::P_{sspB}-*gfp cat*	This study
EBM282	Δ*sigG*::*kan ylnF*::Tn*917*::*amyE*::P_{sigG}-*sigG spc amyE*::P_{sspB}-*gfp cat* Δ*csfB*::tet	This study
EBM287	Δ*sigG*::*kan ylnF*::Tn*917*::*amyE*::$^{15nt,mut7}P_{sigG}$-*ATG*-^{RSS}*sigG* ("$^{quad}P_{sigG}$-*sigG*") *spc amyE*::P_{sspB}-*gfp cat* Δ*csfB*::tet	This study
JC68	*amyE*::P_{sigG}^{+18}-*ATG-lacZ cat*	This study
JC70	*amyE*::$P_{sigG}^{+10\rightarrow+15}$-*ATG-lacZ cat*	This study
JJB31	*amyE*::P_{sigG}-*ATG-comGA*$^{2\text{-}8}$-*lacZ cat*	This study

Strain	Genotype[a,b]	Source or reference
JJB37	*amyE*::P$_{sigG}$^{mut2}-*ATG-comGA*$^{2-8}$-*lacZ cat*	This study
JJB51	*amyE*::^{15nt}P$_{sigG}$-*ATG-comGA*$^{2-8}$-*lacZ cat*	This study
JJB55	*amyE*::P$_{sigG}$^{mut7}-*ATG-comGA*$^{2-8}$-*lacZ cat*	This study
JJB73	*amyE*::P$_{sigG}$-*ATG-comGA*$^{2-8}$-*lacZ cat* Δ*sigG*::*kan*	This study
JJB75	*amyE*::P$_{sigG}$-*ATG-comGA*$^{2-8}$-*lacZ cat* Δ*sigG*::*kan* Δ*sigF*::*erm*	This study
JJB77	*amyE*::P$_{sigG}$-*ATG-comGA*$^{2-8}$-*lacZ cat* Δ*sigG*::*kan* Δ*spoIIIAA-AH*::*erm*	This study
JJB79	*amyE*::P$_{sigG}$-*ATG-comGA*$^{2-8}$-*lacZ cat* Δ*sigG*::*kan* Δ*spoIIQ*::*erm*	This study
JJB85	*amyE*::P$_{sigG}$-*ATG-comGA*$^{2-8}$-*lacZ cat* Δ*sigE-sigG*::*erm*	This study
JJB87	*amyE*::$^{T\rightarrow A}$P$_{sigG}$-*ATG-comGA*$^{2-8}$-*lacZ cat*	This study
JJB89	*amyE*::$^{T\rightarrow G}$P$_{sigG}$-*ATG-comGA*$^{2-8}$-*lacZ cat*	This study
JJB99	*amyE*::$^{15nt,T\rightarrow A}$P$_{sigG}$-*ATG-comGA*$^{2-8}$-*lacZ cat*	This study
JJB101	*amyE*::$^{15nt,T\rightarrow G}$P$_{sigG}$-*ATG-comGA*$^{2-8}$-*lacZ cat*	This study
LMB39	Δ*sigG*::*kan ylnF*::Tn*917*::*amyE*::P$_{sigG}$-*sigG spc*	This study

[a]All *B. subtilis* strains are isogenic with PY79 [62].

[b]For information on the sources of gene deletions, *lacZ* fusions, and other constructs, see “Strain construction” section of S1 Text (Supplemental Materials and Methods).
