# Supplementary material for: Transcription and translation of the sigG gene is tuned for proper execution of the switch from early to late gene expression in the developing Bacillus subtilis spore
Source: PLoS Genet. 2018 Apr 27;14(4):e1007350. doi: 10.1371/journal.pgen.1007350 (PMC5942855; doi:10.1371/journal.pgen.1007350)
Supplement: S2 Table — (PDF) [file pgen.1007350.s008.pdf]

S2 Table. Plasmids used in this study

Plasmid	Description	Source or reference
pAH125	*amyE*::P_{sspB}-*lacZ cat, amp*	This study
pAH158	*amyE*::*gfp cat, amp*	This study
pAH168	*amyE*::*'sigG cat, amp*	[19]
pAH182	*amyE*::P_{sigG}-*sigG' spc, amp*	This study
pAH255	*amyE*::$P_{sigG}{}^{+10\text{-}30}$-*ATG-lacZ cat, amp*	This study
pAH342	*amyE*::P_{sigG}-*ATG-lacZ cat, amp*	This study
pAH485	*amyE*::P_{sspB}-*gfp cat, amp*	This study
pAH606	*amyE*::$P_{sigG}{}^{+24\rightarrow+30}$-*ATG-lacZ cat, amp*	This study
pAH608	*amyE*::$P_{sigG}{}^{+10\rightarrow+18}$-*ATG-lacZ cat, amp*	This study
pAH620	*amyE*::${}^{15nt}P_{sigG}{}^{mut7}$-*ATG-*^{RSS}*sigG'* ("${}^{quad}P_{sigG}$-*sigG'*") *spc, amp*	This study
pAM3	*amyE*::P_{sigG}-*spacer-RBS-ATG-lacZ cat, amp*	This study
pAM4	*amyE*::$P_{sigG}{}^{+10\text{-}15}$-*spacer-RBS-ATG-lacZ cat, amp*	This study
pEBM90	*amyE*::P_{sigG}-*sigG*${}^{1\text{-}28}$-*lacZ cat, amp*	This study
pEBM91	*amyE*::P_{sigG}-*ATG-sigG*${}^{2\text{-}28}$-*lacZ cat, amp*	This study
pEBM94	*amyE*::P_{sigG}-*ATG-*^{RSS}*sigG*${}^{2\text{-}28}$-*lacZ cat, amp*	This study
pEBM99	*amyE*::${}^{15nt}P_{sigG}{}^{mut7}$-*ATG-*^{RSS}*sigG*${}^{2\text{-}28}$-*lacZ* ("${}^{quad}P_{sigG}$-*lacZ*") *cat, amp*	This study
pJC6	*amyE*::$P_{sigG}{}^{+10\text{-}15}$-*ATG-lacZ cat, amp*	This study
pJJ8	*amyE*::P_{sigG}-*ATG-comGA*${}^{2\text{-}8}$-*lacZ cat, amp*	This study
pJJ17	*amyE*::$P_{sigG}{}^{mut2}$-*ATG-comGA*${}^{2\text{-}8}$-*lacZ cat, amp*	This study
pJJ24	*amyE*::*ATG-comGA*${}^{2\text{-}8}$-*lacZ cat, amp*	This study
pJJ26	*amyE*::${}^{15nt}P_{sigG}$-*ATG-comGA*${}^{2\text{-}8}$-*lacZ cat, amp*	This study
pJJ29	*amyE*::$P_{sigG}{}^{mut7}$-*ATG-comGA*${}^{2\text{-}8}$-*lacZ cat, amp*	This study
pJJ38	*amyE*::${}^{T\rightarrow A}P_{sigG}$-*ATG-comGA*${}^{2\text{-}8}$-*lacZ cat, amp*	This study
pJJ39	*amyE*::${}^{T\rightarrow G}P_{sigG}$-*ATG-comGA*${}^{2\text{-}8}$-*lacZ cat, amp*	This study
pJJ44	*amyE*::${}^{15nt,T\rightarrow A}P_{sigG}$-*ATG-comGA*${}^{2\text{-}8}$-*lacZ cat, amp*	This study
pJJ45	*amyE*::${}^{15nt,T\rightarrow G}P_{sigG}$-*ATG-comGA*${}^{2\text{-}8}$-*lacZ cat, amp*	This study
