# Supplementary material for: Transcription and translation of the sigG gene is tuned for proper execution of the switch from early to late gene expression in the developing Bacillus subtilis spore
Source: PLoS Genet. 2018 Apr 27;14(4):e1007350. doi: 10.1371/journal.pgen.1007350 (PMC5942855; doi:10.1371/journal.pgen.1007350)
Supplement: S3 Table — (PDF) [file pgen.1007350.s009.pdf]

S3 Table. Oligonucleotides used in this study

**Oligo**	**Sequence (5’ → 3’)***	**Description**
AH6	gatc**gaattc**agcggatatgatggggatttc	P_{sigG} upstream, forward primer with EcoRI site. Used to construct pAH182, pAH255, pAH342, pAH606, pAH608, pJC5, and pJC6.
AH60	gatc**gaattc**acgagatacatgaactgatgc	P_{sspB} upstream, forward primer with EcoRI site. Used to construct pAH125, pAH485.
AH88	gatc**aagctt**caTGAtAAtTcctccTtaTGTAGActgctgtacaacgttaagtatctcc	P_{sigG} downstream reverse primer with engineered ATG start codon, substitutions spanning the *sigG* leader sequence from positions +10 to +30, and in-frame HindIII site. Used to construct pAH255.
AH96	gatc**aagctt**catgtgtaaaatctcctttttatttagtatgg	P_{sspB} downstream reverse primer with native ATG start codon and in-frame HindIII site. Used to construct pAH125, pAH485.
AH118	gatc**aagctt**agtaaaggagaagaacttttcactggag	*gfp* forward primer with HindIII site. Used to construct pAH185.
AH119	gatc**ggatcc**ttatttgtatagttcatccatgccatgtg	*gfp* reverse primer with engineered stop codon and BamHI site. Used to construct pAH185.
AH121	gatc**gacgtc**tgcggtacttcaagaacttttgcg	*sigG* internal reverse primer with AatII site. Used to construct pAH182.
AH334	gatc**aagctt**caTtttttccctccctacaggagctg	P_{sigG} downstream reverse primer with engineered ATG start codon and in-frame HindIII site. Used to construct pAH342.
AH335	ggtctgagtgcagtgcataAtttttcccacccaagg	Mutagenesis primer to lengthen the spacer between the -35 and -10 *sigG* promoter elements to 15 nt, top strand. Used to construct pJJ26.
AH336	ccttgggtgggaaaaaTtatgcactgcactcagacc	Mutagenesis primer to lengthen the spacer between the -35 and -10 *sigG* promoter elements to 15 nt, bottom strand. Used to construct pJJ26.
AM1	/5’phos/**agct**tgaagacgttgtgttaagacggactgtcgtatgacaagatctaaaggaggaactactatgc	Oligonucleotide harboring spacer and an engineered RBS, top strand. 5’ end was phosphorylated and harbors nucleotides compatible for annealing to HindIII single-stranded overhang. Used to construct pAM3 and pAM4.
AM2	/5’phos/**agct**gcatagtagttcctcctttagatcttgtcatacgacagtccgtcttaacacaacgtcttca	Oligonucleotide harboring spacer and an engineered RBS, bottom strand. 5’ end was phosphorylated and harbors nucleotides compatible for annealing to HindIII single-stranded overhang. Used to construct pAM3 and pAM4.
JJ4	ctattgccgatgataagctgtcaaacatga**gaattc**agcggatatgatggggatttctc	P_{sigG} upstream, forward primer with homology to upstream plasmid sequence in pAH124. Used to construct pJJ8.
JJ5	cgattaagttgggtaacgccagggttttcgcttaccttttctattgaatccatttttccctccctacaggagc	P_{sigG} downstream reverse primer with ATG start codon, *comGA* codons 2-8, and homology to *lacZ* in pAH124. Used to construct pJJ8.

Oligo	Sequence (5' → 3')*	Description
JJ8	cgttgtacagTCCctcctgtagggag	Mutagenesis primer to insert the "*mut2*" mutation into the *sigG* leader sequence, top strand, used to construct pJJ17.
JJ9	ccctacaggagGGActgtacaacgttaagtatc	Mutagenesis primer to insert the "*mut2*" mutation into the *sigG* leader sequence, bottom strand. Used to construct pJJ17.
JJ14	ctattgccgatgataagctgtcaaacatga**gaattc**gat**aagctt**ATGGATTCAATAGAAAAGGTAAGCgaaaaccctggcgttacccaacttaatcgc	Oligonucleotide harboring EcoRI and HindIII sites, ATG start codon, *comGA* codons 2-8 single-stranded overhang, and flanking sequences homologous to pAH124, top strand. Used to construct pJJ24.
JJ15	gcgattaagttgggtaacgccagggttttcGCTTACCTTTTCTATTGAATCCAT**aagctt**atc**gaattc**tcatgtttgacagcttatcatcggcaatag	Oligonucleotide harboring EcoRI and HindIII sites, ATG start codon, *comGA* codons 2-8 single-stranded overhang, and flanking sequences homologous to pJJ8, bottom strand. Used to construct pJJ24.
JJ20	gttgtacagcAGAgactgtagggagggaaaaaaatgg	Mutagenesis primer to insert the "*mut7*" mutation into the *sigG* leader sequence, top strand. Used to construct pJJ29.
JJ21	ccatttttttccctccctacagtcTCTgctgtacaac	Mutagenesis primer to insert the "*mut7*" mutation into the *sigG* leader sequence, bottom strand. Used to construct pJJ29.
oAC20	gatcaagcttcaTGAtAAtTcctccctacaggagctgctg	P_{sigG} downstream reverse primer with engineered ATG start codon, substitutions spanning the *sigG* leader sequence from positions +24 to +30, and in-frame HindIII site, used to construct pAH606
oAC21	gatcaagcttcaTttttttccctccTtaTGTAGActgctgtacaacgttaagtatctcc	P_{sigG} downstream reverse primer with engineered ATG start codon, substitutions spanning the *sigG* leader sequence from positions +10 to +18, and in-frame HindIII site, used to construct pAH608
oAC23	gatcaagcttcaTttttttccctccTtacaggagctgctgtacaacgttaagtatctcc	P_{sigG} downstream reverse primer with engineered ATG start codon, a substitution in the *sigG* leader sequence at position +18, and in-frame HindIII site, used to construct pJC5
oAC24	gatc**aagctt**caTttttttccctccctaTGTAGActgctgtacaacgttaagtatctcc	P_{sigG} downstream reverse primer with engineered ATG start codon, substitutions spanning the *sigG* leader sequence from positions +10 to +15, and in-frame HindIII site, used to construct pJC6.

*Relevant restriction endonuclease recognition sites and/or sequences that provide complementarity to restriction site single-stranded overhangs are in bold. Relevant substitutions or insertions in mutagenesis primers are indicated in uppercase. Sequences that provide complementarity to target plasmids are underlined.
