# Supplementary material for: Transcription and translation of the sigG gene is tuned for proper execution of the switch from early to late gene expression in the developing Bacillus subtilis spore
Source: PLoS Genet. 2018 Apr 27;14(4):e1007350. doi: 10.1371/journal.pgen.1007350 (PMC5942855; doi:10.1371/journal.pgen.1007350)
Supplement: S4 Table — (PDF) [file pgen.1007350.s010.pdf]

S4 Table. Synthetic DNA fragments used in this study

gBlock	Sequence*	Description
gJJ2	ccgatgataagctgtcaaacatgagaattcagcggatatgatgggg<br>atttctcagtcttatatttcgcggcttgagaaaagaattataaaaaggtt<br>gagaaaagagttcaacaaaatggtgtaaaaaattttatggttagaac<br>cccttgattttacagggatttcctgatttcgacagtttttcggtctgagtgc<br>agtgcatatttttcccacccaaggagatactAaacgttgtacagcagc<br>tcctgtagggagggaaaaaaatggattcaatagaaaaggtaagcg<br>aaaa	Harbors *sigG* promoter and RBS, with T→A substitution at position -7, and flanked by sequences homologous to pJJ24. Used to make pJJ38.
gJJ3	ccgatgataagctgtcaaacatgagaattcagcggatatgatgggg<br>atttctcagtcttatatttcgcggcttgagaaaagaattataaaaaggtt<br>gagaaaagagttcaacaaaatggtgtaaaaaattttatggttagaac<br>cccttgattttacagggatttcctgatttcgacagtttttcggtctgagtgc<br>agtgcatatttttcccacccaaggagatactGaacgttgtacagcagc<br>tcctgtagggagggaaaaaaatggattcaatagaaaaggtaagcg<br>aaaa	Harbors *sigG* promoter and RBS, with T→G substitution at position -7, and flanked by sequences homologous to pJJ24. Used to make pJJ39.
gJJ6	ccgatgataagctgtcaaacatgagaattcagcggatatgatgggg<br>atttctcagtcttatatttcgcggcttgagaaaagaattataaaaaggtt<br>gagaaaagagttcaacaaaatggtgtaaaaaattttatggttagaac<br>cccttgattttacagggatttcctgatttcgacagtttttcggtctgagtgc<br>agtgcataAtttttcccacccaaggagatactAaacgttgtacagca<br>gctcctgtagggagggaaaaaaatggattcaatagaaaaggtaag<br>cgaaaa	Harbors *sigG* promoter and RBS, with T→A substitution at position -7 and lengthened promoter spacer (increased to 15 nt), and flanked by sequences homologous to pJJ24. Used to make pJJ44.
gJJ7	ccgatgataagctgtcaaacatgagaattcagcggatatgatgggg<br>atttctcagtcttatatttcgcggcttgagaaaagaattataaaaaggtt<br>gagaaaagagttcaacaaaatggtgtaaaaaattttatggttagaac<br>cccttgattttacagggatttcctgatttcgacagtttttcggtctgagtgc<br>agtgcataAtttttcccacccaaggagatactGaacgttgtacagca<br>gctcctgtagggagggaaaaaaatggattcaatagaaaaggtaag<br>cgaaaa	Harbors *sigG* promoter and RBS, with T→G substitution at position -7 and lengthened promoter spacer (increased to 15 nt), and flanked by sequences homologous to pJJ24. Used to make pJJ45.
gAC2	aaatcgccattcgccagggctgcaggaattcagcggatatgatggg<br>gatttctcagtcttatatttcgcggcttgagaaaagaattataaaaaggt<br>tgagaaaagagttcaacaaaatggtgtaaaaaattttatggttagaac<br>cccttgattttacagggatttcctgatttcgacagtttttcggtctgagtgc<br>agtgcataAtttttcccacccaaggagatacttaacgttgtacagcag<br>AGActgtagggagggaaaaaaAtgtcAagaaaCaaagtAgaa<br>atAtgcggAgtAgaCacAtcAaaaCtaccagtactAaaAaaCg<br>aagaAatgagaaaActAttCaggcagctgcaggatgaaggcgat<br>gattcagcaagagaaaagcttgtaaacgggaacttgcgtcttgtct	Harbors *sigG* promoter, RBS, and *sigG* codons 1-51, flanked by sequences homologous to pAH182. Sequence was engineered to lengthen spacer between -35 and -10 *sigG* promoter elements, introduce “mut7” mutation in *sigG* leader sequence, switch *sigG* start codon to ATG, and reduce secondary structure in *sigG* codons 2-28. Used to make pAH620.
gEBM10	gataagctgtcaaacatgagaattcagcggatatgatggggatttctc<br>agtcttatatttcgcggcttgagaaaagaattataaaaaggttgagaa<br>aagagttcaacaaaatggtgtaaaaaattttatggttagaacccccttg<br>attttacagggatttcctgatttcgacagtttttcggtctgagtgcagtgca<br>tatttttcccacccaaggagatacttaacgttgtacagcagctcctgtag<br>ggagggaaaaaagtgtcgagaaataaagtcgaaatctgcggggtg<br>gatacctccaaattaccagtactcaagaatgaagagatgagaaagc<br>tgtttgaaaaccctggcgttacccaactta	Harbors *sigG* promoter, RBS, and *sigG* codons 1-28, flanked by sequences homologous to pAH124. Used to make pEBM90.
gEBM11	gataagctgtcaaacatgagaattcagcggatatgatggggatttctc<br>agtcttatatttcgcggcttgagaaaagaattataaaaaggttgagaa<br>aagagttcaacaaaatggtgtaaaaaattttatggttagaacccccttg<br>attttacagggatttcctgatttcgacagtttttcggtctgagtgcagtgca<br>tatttttcccacccaaggagatacttaacgttgtacagcagctcctgtag<br>ggagggaaaaaaAtgtcgagaaataaagtcgaaatctgcggggt<br>ggatacctccaaattaccagtactcaagaatgaagagatgagaaag<br>ctgtttgaaaaccctggcgttacccaactta	Harbors *sigG* promoter, RBS, and *sigG* codons 2-28, flanked by sequences homologous to pAH124. Sequence was engineered to switch *sigG* start codon to ATG. Used to make pEBM91.

gBlock	Sequence*	Description
gEBM13	gataagctgtcaaacatgagaattcagcggatatgatggggatttctcagtcttatatttcgcggcttgagaaaagaattataaaaaggttgagaaaagagttcaacaaaatggtgtaaaaaattttatggttagaaccccttgattttacagggatttcctgatttcgacagtttttcggtctgagtgcagtgcatatttttcccacccaaggagatacttaacgttgtacagcagctcctgtagggagggaaaaaaAtgtcAagaaaCaaagtAgaaatAtgcggAgtAgaCacAtcAaaaCtaccagtactAaaAaaCgaagaAatgagaaaActAttCgaaaaccctggcgttacccaactta	Harbors *sigG* promoter, RBS, and *sigG* codons 2-28, flanked by sequences homologous to pAH124. Sequence was engineered to switch *sigG* start codon to ATG and reduce secondary structure in *sigG* codons 2-28. Used to make pEBM94.
gEBM14	ccgatgataagctgtcaaacatgagaattcagcggatatgatggggatttctcagtcttatatttcgcggcttgagaaaagaattataaaaaggttgagaaaagagttcaacaaaatggtgtaaaaaattttatggttagaaccccttgattttacagggatttcctgatttcgacagtttttcggtctgagtgcataAtttttcccacccaaggagatacttaacgttgtacagcagAGActgtagggagggaaaaaaAtgtcAagaaaCaaagtAgaaatAtgcggAgtAgaCacAtcAaaaCtaccagtactAaaAaaCgaagaAatgagaaaActAttCgaaaaccctggcgttacccaactt	Harbors *sigG* promoter, RBS, and *sigG* codons 2-28, flanked by sequences homologous to pAH124. Engineered to incorporate the following alterations: insertion to lengthen the spacer between *sigG* -35 and -10 promoter elements, “mut7” mutation in *sigG* leader sequence, switch *sigG* start codon to ATG, and reduced secondary structure substitutions in *sigG* codons 2-28. Used to make pEBM99.

*Sequences that provide complementarity to target plasmids are underlined. Relevant substitutions or insertions are indicated in uppercase.
