# Supplementary material for: Transcription and translation of the sigG gene is tuned for proper execution of the switch from early to late gene expression in the developing Bacillus subtilis spore
Source: PLoS Genet. 2018 Apr 27;14(4):e1007350. doi: 10.1371/journal.pgen.1007350 (PMC5942855; doi:10.1371/journal.pgen.1007350)
Supplement: S1 Text — (PDF) [file pgen.1007350.s011.pdf]

S1 Text. Supplemental materials and methods

Strain construction

All *B. subtilis* strains were isogenic with the laboratory strain PY79 [62]. Derivatives of PY79 were generated by transformation with chromosomal DNA from *B. subtilis* or plasmid DNA. Competent *B. subtilis* cells were prepared as previously described [70]. Selection on media with antibiotics, when appropriate, was performed as follows: chloramphenicol (5 μg/mL), erythromycin plus lincomycin (1 μg/mL and 25 μg/mL, respectively), spectinomycin (100 μg/mL), kanamycin (5 μg/mL), tetracycline (10 μg/mL). Chromosomal integration into the *amyE* locus was confirmed by loss of α-amylase activity on LB agar plates with starch; insertion into the "alternative *amyE*" site at *ylnF* was as previously described [13,19]. The full genotypes of strains used in this study are given in S1 Table. The antibiotic resistance genes utilized to confer resistance to antibiotics are referred to as follows: *cat* (chloramphenicol), *erm* (erythromycin plus lincomycin), *spc* (spectinomycin), *kan* (kanamycin), and *tet* (tetracycline). Plasmids used in this study are listed in S2 Table and details of plasmid design and construction are provided in the "Plasmid construction" section below.

Deletion mutants. The Δ*sigG*::*kan*, Δ*spoIIQ*::*erm*, Δ*csfB*::*tet*, and Δ*sigE-sigG*::*erm* deletions were obtained from strains AHB98, AHB141, AHB199, and AHB400, respectively, which were previously described [19]. The Δ*sigF*::*erm* deletion was obtained from strain RL1275 [71], which was built by moving Δ*sigF*::*erm* from MO173 (gift of P. Stragier) into PY79. The Δ*spoIIIAA-AH*::*erm* deletion was obtained

from strain RL2765 (gift of R. Losick), which was built by moving Δ*spoIIIAA-AH*::*erm* from MO1433 (gift of P. Stragier) into PY79.

***lacZ* reporter constructs.** The *lacZ* reporter constructs used in this study were integrated at the *amyE* locus. The P_{spoIIQ}-*lacZ* reporter construct was previously described [13]. All remaining *lacZ* reporter constructs were built for this study (see "Plasmid construction" section below).

***sigG* constructs.** Wild type and mutant *sigG* variants were inserted at the *ylnF* "alternative *amyE*" locus [13] in a two-step process due to toxicity of full-length *sigG* in *Escherichia coli*, as has been previously reported [9]. First, the 3' end of the *sigG* coding sequence was integrated using plasmid pAH168 (*amyE*::*'sigG cat, amp*) [19]. The remainder of *sigG*, including upstream regulatory sequences, was then inserted in a second transformation event using either plasmid pAH182 (*amyE*::P_{sigG}-*sigG' spc, amp*) or plasmid pAH620 (*amyE*:: $^{15nt,mut7}P_{sigG}$-*ATG*-^{RSS}*sigG' spc, amp*), which were built for this study (see "Plasmid construction" section below).

Plasmid construction

Plasmids used in this study are listed in S2 Table. The sequences of oligonucleotides used in plasmid construction are given in S3 Table. Chromosomal DNA from PY79 served as a template for PCR, unless otherwise noted. Synthetic gene fragments (gBlocks, IDT) used in plasmid construction are listed in S4 Table. Plasmids were constructed using traditional cloning techniques, site-directed mutagenesis, or isothermal assembly [72], as indicated, and were propagated in the *E. coli* strains DH5α

or NEB 5-alpha (NEB). All plasmids were verified by DNA sequencing. Plasmids not previously published were constructed as follows:

pAH125 (*amyE*::P_{sspB}-*lacZ cat, amp*) was constructed by ligating a HindIII-digested PCR product containing the *sspB* promoter, RBS, and start codon (amplified with AH60 and AH96) into HindIII-digested pAH124 (*amyE*::*lacZ cat, amp*) [13].

pAH182 (*amyE*::P_{sigG}-*sigG' spc, amp*) was constructed by ligating an EcoRI/AatII PCR product containing the 5' region of the *sigG* coding sequence and upstream *sigG* promoter sequences (amplified with AH6 and AH121) into the EcoRI/AatII-digested backbone of pDG1730 (*amyE*::*spc, amp*) [73].

pAH255 (*amyE*::$P_{sigG}^{+10\text{-}30}$-*ATG-lacZ cat, amp*) was constructed by ligating an EcoRI/HindIII-digested PCR product harboring the *sigG* promoter and RBS (amplified with AH6 and AH88) into EcoRI/BamHI-digested pAH124 (*amyE*::*lacZ cat, amp*) [13]. The mutagenic primer AH88 introduced an ATG start codon in place of the native *sigG* GTG start codon, as well as 12 additional substitutions upstream and downstream of the RBS (spanning positions +10 through +30).

pAH342 (*amyE*::P_{sigG}-*ATG-lacZ cat, amp*) was constructed by ligating an EcoRI/HindIII-digested PCR product harboring the *sigG* promoter and RBS (amplified with AH6 and AH334) into EcoRI/BamHI-digested pAH124 (*amyE*::*lacZ cat, amp*) [13]. The mutagenic primer AH334 introduced an ATG start codon in place of the native *sigG* GTG start codon.

pAH485 (*amyE*::P_{sspB}-*gfp cat, amp*) was constructed in two steps. First, a HindIII/BamHI-digested PCR product harboring the "mut2" variant of *gfp* (amplified with

AH118 and AH119 from pAC172 [74]) was ligated into HindIII/BamHI-digested pDG1662 (*amyE*::*cat, amp*) [73], yielding **pAH158** (*amyE*::*gfp cat, amp*). Second, a HindIII-digested PCR product containing the *sspB* promoter, RBS, and start codon (amplified with AH60 and AH96) was ligated into HindIII-digested pAH158, yielding pAH485.

pAH606 (*amyE*::$P_{sigG}^{+24\text{-}30}$-*ATG-lacZ cat, amp*), **pAH608** (*amyE*::$P_{sigG}^{+10\text{-}18}$-*ATG-lacZ cat, amp*), **pJC5** (*amyE*::P_{sigG}^{+18}-*ATG-lacZ cat, amp*), and **pJC6** (*amyE*::$P_{sigG}^{+10\text{-}15}$-*ATG-lacZ cat, amp*) were designed to harbor different subsets of the mutations present in pAH255 (*amyE*::$P_{sigG}^{+10\text{-}30}$-*ATG-lacZ cat, amp*). Each was constructed by ligating an EcoRI/HindIII-digested PCR product with the appropriate suite of substitutions into EcoRI/HindIII-digested pAH124 (*amyE*::*lacZ cat, amp*) [13]. Primer pairs used for PCR amplification were as follows: AH6/oAC20 (pAH606), AH6/oAC21 (pAH608), AH6/oAC23 (pJC5), and AH6/oAC24 (pJC6).

pAH620 (*amyE*:: ${}^{15nt,mut7}P_{sigG}$-*ATG-*^{RSS}*sigG' spc, amp*) was constructed by isothermal assembly of a synthetic DNA fragment (gAC2) harboring the 5' region of the *sigG* coding sequence and upstream *sigG* promoter sequences, with relevant alterations and flanked by homology to the target plasmid, into the EcoRI/HindIII-digested backbone of pAH182 (*amyE*::P_{sigG}-*sigG' spc, amp*).

pAM3 (*amyE*::P_{sigG}-*spacer-RBS-ATG-lacZ cat, amp*) and **pAM4** (*amyE*::$P_{sigG}^{+10\text{-}15}$-*spacer-RBS-ATG-lacZ cat, amp*) were constructed by ligating a DNA fragment (generated by annealing oligonucleotides AM1 and AM2) harboring a spacer followed by an engineered RBS into HindIII-digested pAH342 (*amyE*::P_{sigG}-*ATG-lacZ cat, amp*) or pJC6 (*amyE*::$P_{sigG}^{+10\text{-}15}$-*ATG-lacZ cat, amp*), respectively.

pEBM90 (*amyE*::P_{sigG}-*sigG*$^{1\text{-}28}$-*lacZ cat, amp*), **pEBM91** (*amyE*::P_{sigG}-*ATG-sigG*$^{2\text{-}28}$-*lacZ cat, amp*), **pEBM94** (*amyE*::P_{sigG}-*ATG*-^{RSS}*sigG*$^{2\text{-}28}$-*lacZ cat, amp*), and **pEBM99** (*amyE*::$^{15nt,mut7}\mathrm{P}_{sigG}$-*ATG*-^{RSS}*sigG*$^{2\text{-}28}$-*lacZ cat, amp*) were constructed by isothermal assembly of synthetic DNA fragments (gEBM10, gEBM11, gEBM13, or gEBM14, respectively) harboring the first 28 *sigG* codons and upstream *sigG* promoter sequences, with relevant alterations and flanked by homology to the target plasmid, into the EcoRI-digested backbone of pAH124 (*amyE*::*lacZ cat, amp*) [13].

pJJ8 (*amyE*::P_{sigG}-*ATG-comGA*$^{2\text{-}8}$-*lacZ cat, amp*) was constructed by PCR amplifying (using primers JJ4 and JJ5) a DNA fragment harboring the *sigG* promoter and RBS, followed by an ATG codon and codons 2-8 from the highly expressed *B. subtilis* gene *comGA*. This fragment, which was also flanked by sequences homologous to the target plasmid, was then inserted into EcoRI/HindIII-digested pAH124 (*amyE*::*lacZ cat, amp*) [13] by isothermal assembly.

pJJ17 (*amyE*::$^{mut2}\mathrm{P}_{sigG}$-*ATG-comGA*$^{2\text{-}8}$-*lacZ cat, amp*), **pJJ26** (*amyE*::$^{15nt}\mathrm{P}_{sigG}$-*ATG-comGA*$^{2\text{-}8}$-*lacZ cat, amp*), and **pJJ29** (*amyE*::$^{mut7}\mathrm{P}_{sigG}$-*ATG-comGA*$^{2\text{-}8}$-*lacZ cat, amp*), were constructed by performing QuikChange mutagenesis (Agilent Technologies) of plasmid pJJ8 (*amyE*::P_{sigG}-*ATG-comGA*$^{2\text{-}8}$-*lacZ cat, amp*) with primers JJ8/JJ9, AH335/AH336, and JJ20/JJ21, respectively.

pJJ38 (*amyE*::$^{T\rightarrow A}\mathrm{P}_{sigG}$-*ATG-comGA*$^{2\text{-}8}$-*lacZ cat, amp*), **pJJ39** (*amyE*:: $^{T\rightarrow G}\mathrm{P}_{sigG}$-*ATG-comGA*$^{2\text{-}8}$-*lacZ cat, amp*), **pJJ44** (*amyE*::$^{15nt,T\rightarrow A}\mathrm{P}_{sigG}$-*ATG-comGA*$^{2\text{-}8}$-*lacZ cat, amp*), and **pJJ45** (*amyE*::$^{15nt,T\rightarrow G}\mathrm{P}_{sigG}$-*ATG-comGA*$^{2\text{-}8}$-*lacZ cat, amp*) were constructed in two steps. First, a DNA fragment (generated by annealing oligonucleotides JJ14 and JJ15) haboring EcoRI and HindIII restriction sites, an ATG start codon, and *comGA*

codons 2-8, was inserted into EcoRI/HindIII-digested pAH124 (*amyE*::*lacZ cat, amp*) [13] by isothermal assembly, yielding **pJJ24** (*amyE*::*ATG-comGA*$^{2\text{-}8}$*-lacZ cat, amp*). Next, synthetic DNA fragments (gJJ2, gJJ3, gJJ6, and gJJ7) harboring relevant alterations and flanked by homology to the target plasmid, were inserted by isothermal assembly into EcoRI/HindIII-digested pJJ24, yielding pJJ38, pJJ39, pJJ44, and pJJ45, respectively.

Additional Supplemental References

70. Wilson GA, Bott KF. Nutritional factors influencing the development of competence in the *Bacillus subtilis* transformation system. J Bacteriol. 1968; 95: 1439–1449. PMID: 4967198

71. Eichenberger P, Fawcett P, Losick R. A three-protein inhibitor of polar septation during sporulation in *Bacillus subtilis*. Mol Microbiol. 2001; 42: 1147–1162. doi: 10.1046/j.1365-2958.2001.02660.x PMID: 11886548

72. Gibson DG, Young L, Chuang R-Y, Venter JC, Hutchison CA, Smith HO. Enzymatic assembly of DNA molecules up to several hundred kilobases. Nat Methods. 2009; 6: 343–345. doi: 10.1038/nmeth.1318 PMID: 19363495

73. Guérout-Fleury AM, Frandsen N, Stragier P. Plasmids for ectopic integration in *Bacillus subtilis*. Gene. 1996; 180: 57–61. doi: 10.1016/S0378-1119(96)00404-0 PMID: 8973347

74. Chastanet A, Losick R. Engulfment during sporulation in *Bacillus subtilis* is governed by a multi-protein complex containing tandemly acting autolysins. Mol Microbiol. 2007; 64: 139–152. doi: 10.1111/j.1365-2958.2007.05652.x PMID: 17376078
